# Harnessing TCR repertoires: predictive insights and therapeutic monitoring in cancer immunotherapy

**DOI:** 10.1016/j.iotech.2025.101076

**Published:** 2025-10-01

**Authors:** J. Chiffelle, R. Genolet, O. Michielin, A. Harari

**Affiliations:** 1 Ludwig Institute for Cancer Research, Lausanne Branch, Department of Oncology, Lausanne University Hospital (CHUV) and University of Lausanne (UNIL), Lausanne, Switzerland; 2 Agora Cancer Research Center, Lausanne, Switzerland; 3 Swiss Cancer Center Léman (SCCL), Lausanne, Switzerland; 4 Department of Oncology, Geneva University Hospitals (HUG) and Department of Medicine, University of Geneva (UNIGE), Geneva, Switzerland

**Keywords:** T-cell receptors, immune repertoire, repertoire diversity, repertoire clonality, biomarker, cancer immunotherapy, immune profiling

## Abstract

The T-cell receptor (TCR) repertoire, representing the vast diversity of T cells, is a cornerstone of adaptive immunity and a powerful tool in oncology. Advances in high-throughput sequencing have enabled deep profiling of TCR diversity and clonality, highlighting the repertoire as a promising biomarker for cancer diagnosis, prognosis and therapeutic monitoring. This review synthesizes the current understanding of TCR repertoire analysis in cancer care. Distinct TCR features in tumors and peripheral blood can differentiate cancer patients from healthy individuals and help stage disease. Prognostically, a focused, clonal intratumoral repertoire is often associated with improved survival, whereas high diversity in peripheral blood typically reflects robust immune competence and better outcomes. In cancer immunotherapy, TCR profiling offers predictive insights; high baseline tumor clonality frequently correlates with response to anti-programmed cell death protein 1/programmed death-ligand 1 inhibitors, while greater peripheral diversity may predict benefit from anti-cytotoxic T-lymphocyte-associated protein 4 (anti-CTLA-4) therapy. Dynamic monitoring often shows an increase in clonality in patients responding to treatment. Furthermore, TCR analysis is integral to optimizing and tracking adoptive cell therapies and cancer vaccines. Despite this potential, significant challenges, including a lack of methodological standardization, currently limit widespread clinical application. Integrating TCR analysis with multi-omic and single-cell technologies is essential to overcoming these hurdles and advancing personalized immunotherapy.

## Highlights

• Blood TCR diversity predicts systemic immune competence and patient prognosis.
• High TIL clonality often correlates with better tumor-targeted antitumor response.
• TCR repertoire dynamics track therapy response in immunotherapy.
• Integrated TCR repertoire analysis offers potential for personalized cancer treatment decisions.

## Introduction

The adaptive immune system is a major achievement of evolution, enabling precise recognition and elimination of pathogens and abnormal cells. Its reach extends far beyond infectious threats, influencing autoimmunity and transplant rejection, and acting as a formidable barrier against cancer, principally through T cells and the diversity of T-cell receptors (TCRs). These receptors are cell-surface, disulfide-linked heterodimers, primarily of the α/β type, with γ/δ variants present in ∼1%-5% of T cells.^1^ During thymic maturation, TCR loci undergo V(D)J recombination, randomly assembling gene segments to generate extensive diversity. This process creates three complementarity-determining regions (CDR1-3), with the hypervariable CDR3, shaped by imprecise recombination, interacting, together with CDR1 and CDR2, with peptides presented by major histocompatibility complexes (MHCs).^2^ The diversity of TCRs is crucial for the immune system’s optimal function, including the detection of tumor-associated antigens and tumor-specific antigens in cancer.

Fueled by advances in next-generation sequencing (NGS), each individual’s unique portfolio of TCRs can now be decoded at unprecedented depth, revealing previously hidden layers of immune diversity and clonal evolution.^3^ The recent convergence of single-cell sequencing and RNA-profiling technologies has propelled the field even further, enabling granular mapping of TCR diversity while simultaneously capturing cellular states and functional phenotypes in real time.^3^ These innovations have deepened our understanding of how immunity interacts with tumor evolution and are powering a new era of precision cancer therapies and dynamic biomarkers.^3^^,^^4^

**Figure 1:**
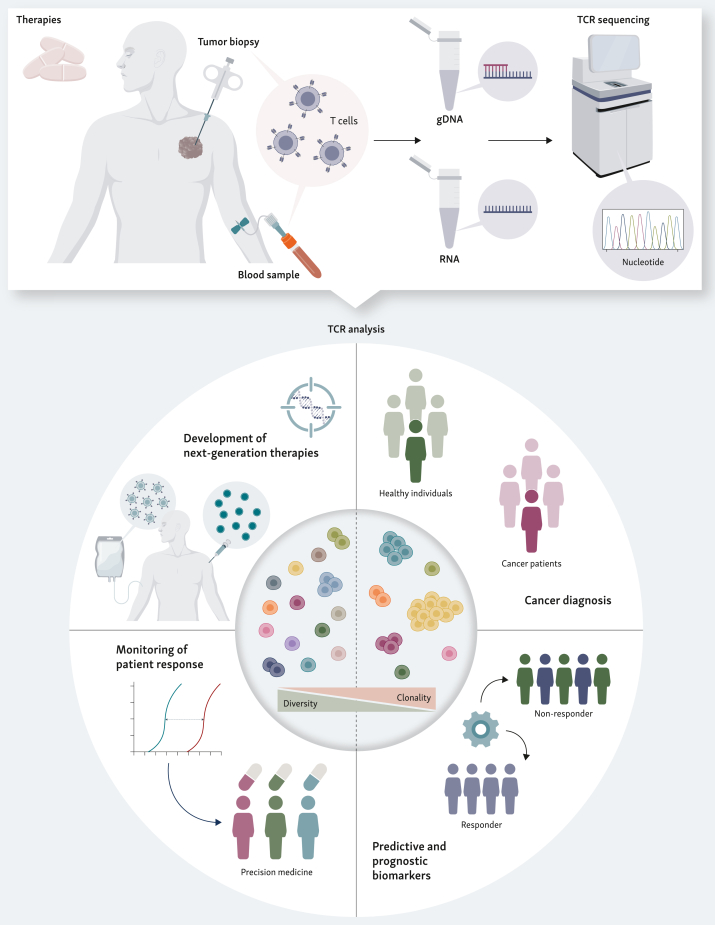
**TCR sequencing empowers personalized cancer care.** In cancer patients, TCRs are profiled using next-generation sequencing from DNA or RNA isolated from blood or tumor samples. Sequencing and computational analysis yield comprehensive insights into the T-cell repertoire, allowing the distinction between healthy individuals and cancer patients, patient stratification, predictive and prognostic biomarker development, and longitudinal monitoring of treatment response to support timely and tailored therapy decisions, such as guiding the development of next-generation therapies. Created with BioRender.com. gDNA, genomic DNA; TCR, T-cell receptor.

Over the past decades, cancer care has undergone a paradigm shift, moving from conventional modalities to personalized approaches, including immune checkpoint inhibitors (ICIs), cellular therapies and cancer vaccines that harness the immune system, particularly T cells. Yet, clinical responses remain limited to a subset of patients.^5^ This challenge fuels the search for robust biomarkers, with mounting evidence that T-cell repertoire features can predict clinical benefit to immunotherapy and guide treatment decisions.^3^^,^^4^ Schematically, immune repertoires can be represented along a spectrum: at one end, high diversity, with many distinct clonotypes, often reflects robust immune fitness; at the other, a biased and clonal repertoire, dominated by a few clonotypes, is typically associated with dominant antigen-specific T-cell responses. Comprehensive TCR profiling thus emerges as a powerful tool to capture immune status and inform diagnosis and prognosis, to stratify patients, and to unlock the full potential of personalized cancer care. In this review, we spotlight how TCR repertoire profiling informs cancer diagnosis, disease evolution and therapeutic monitoring, most notably in the context of immunotherapy, while highlighting its broader promise as a predictive biomarker for treatment response. Decoding the TCR repertoire is not just a technological achievement, it is reshaping the clinical landscape and opening new frontiers in personalized care in cancer and beyond (Figure 1).

## Approaches to TCR sequencing and analysis

### Evolution and challenges of TCR repertoire methodologies

Capturing the full complexity and vast diversity of immune repertoires has long challenged immunologists. Early methods, such as spectratyping providing insights into CDR3 size variability and distributions or Sanger sequencing, offered limited insights and throughput.^3^ The advent of NGS enabled the simultaneous sequencing of millions of TCRs, profoundly improving our understanding of repertoire diversity and clonal distribution.^3^^,^^6^ However, as with any new technology, NGS introduced its share of challenges starting with the material choice for amplification: DNA or RNA (Figure 1). While DNA offers greater stability, RNA techniques are more sensitive due to the higher copy number per cell.^6^ Although RNA was once thought to introduce quantification bias due to variations in TCR expression, single-cell analyses have disproved this concern.^7^

Regardless of the starting material, the central challenge remains amplifying TCRs in a way that fully and accurately captures repertoire diversity, without distorting clonotype representation. Straightforward and cost-effective techniques like multiplex polymerase chain reaction (PCR), using a mix of primers for V and J segments, remain widely used for both DNA and RNA, but differing primer efficiency can lead to selective biased amplification.^6^ While bioinformatics tools and internal controls help mitigate this issue, sequences that would unsuccessfully be amplified due to primer inefficiency cannot be recovered, resulting in a biased and distorted representation of the true repertoire.^8^ The rapid amplification of cDNA ends (5′-RACE) method circumvents some primer bias by using a universal adapter, although its efficiency is hindered by the limited success rate of template switching, resulting in adapter addition to only 20%-60% of RNA molecules.^9^^,^^10^ Despite these limitations, bulk TCR sequencing approaches have substantially advanced the field, providing critical insights into T-cell diversity, clonotype dynamics and immune responses, laying the foundation for more sensitive and accurate methods. To address the limitations of both multiplex PCR and 5′-RACE, innovations like SEQuencing T cell receptor (SEQTR), which combines *in vitro* transcription with multiplex reverse transcription, enhances repertoire sensitivity and accuracy, providing a more reliable approach to studying TCR diversity, resulting in a higher resolution depth than the leading providers of immune profiling.^7^

However, bulk TCR sequencing technologies have historically faced limitations in linking both receptor chains, as they are amplified and sequenced independently. Single-cell RNA sequencing and TCR sequencing overcome this by simultaneously capturing both chains and linking them to each cell’s transcriptomic profile. While early single-cell approaches were restricted by throughput,^11^ advances in unique molecular identifier and microfluidics now allow profiling of up to millions of cells.^10^^,^^12^^,^^13^ While the high cost of single-cell analyses remains a significant barrier to their widespread application in large patient cohorts, approaches such as PairSEQ^14^ or more recently Throughput-Intensive Rapid TCR Library sequencing (TIRTL-seq)^15^ used combinatorial strategies instead of physical isolation to identify α/β TCRs in a cost-effective way. Furthermore, computational tools such as MiXCR^16^ also enable extraction of TCR data directly from RNA sequencing datasets.

### Analytical and computational tools for TCR data interpretation

Immune profiling generates massive datasets that must be distilled into interpretable metrics. Beyond simple clonotype enumeration, advanced ecological measures are used to characterize TCR repertoire complexity.^10^ Key parameters include diversity, encompassing richness (number of unique clonotypes) and evenness (clonal distribution), and clonality, which reflects repertoire dominance by one or a few expanded clones. Numerous indices exist, each highlighting different aspects of repertoire complexity, with no single gold standard.^10^ Reliance on a single index can be misleading, especially given sampling biases and the nonlinear behavior of these measures. As a result, careful interpretation and the simultaneous analysis of multiple indices and metrics are essential for robust and comparable immune profiling.^10^ Tracking changes in diversity, clonality, repertoire overlap, and clonotype trajectory provides valuable insights into immune dynamics and patient monitoring.

Despite advances, predicting which receptors bind to which antigens remain a major challenge due to the huge complexity and cross-reactivity of TCR : peptide-MHC (pMHC) interactions. Achieving this could revolutionize cancer detection and monitoring. *In silico* tools, such as NetMHCpan^17^ and MixMHCpred,^18^ use machine learning trained on extensive epitope databases to estimate how likely a peptide is to bind an MHC molecule, while other algorithms tend to predict TCR specificity for given pMHC complexes. A first group of algorithms, including Grouping of Lymphocyte Interactions by Paratope Hotspots (GLIPH),^19^ TCRDist,^20^ ClusTCR^21^ or Geometric Isometry-based TCR AligNment Algorithm (GIANA),^22^ cluster TCRs by sequence similarity to infer shared antigen specificity. When patients’ TCRs are predicted to group with others known to recognize viral or cancer antigens, this may suggest an ongoing immune response against those antigens and thus the underlying pathology. More sophisticated tools like NetTCR^23^ and MixTCRpred^24^ leverage machine learning to directly predict TCR–pMHC binding, though their accuracy remains questioned, partially due to limitations in training databases like IMGT or VDJdb.^25^ A third category of tools, such as TCRDock^26^ and TCRpcDist,^27^ model the three-dimensional structure of the TCR–pMHC complex using protein structure prediction. However, despite major development in these fields, current tools fail to predict pMHC binding to unseen targets. Despite the limitations in precision, reliability and scalability of current algorithms,^25^ these technologies are ushering in a new era of ‘TCR biology 3.0’, where multi-layered insights, encompassing TCR sequence diversity, structural conformations, human leukocyte antigen (HLA) restrictions, and cross-reactivity, are integrated to deepen our understanding of T-cell immunity of cancer and therapy.

## T-cell repertoires in cancer patients

### TCR repertoire profiles as potential diagnostic tools

A growing body of literature highlights TCR repertoire features as powerful biomarkers for cancer, with high-throughput sequencing enabling detailed profiling in both tumor and blood (Figure 1).^3^^,^^4^ TCR repertoire profiling was reported to distinguish malignant from healthy tissues, stage cancer progression and evaluate ‘hot’ (immunologically active) versus ‘cold’ (immunologically inert) tumor status.^3^^,^^4^ However, TCR landscapes are not only shaped by disease but also by host factors such as age, sex, gut microbiome, inflammation or smoking habits, for example.^28^^,^^29^ Notably, TCR diversity declines with age both in healthy individuals and cancer patients,^29^, ^30^, ^31^ reflecting thymic involution.^29^ Smoking habits (all tobacco smokers) also affect immune repertoire by reducing repertoire richness and diversity in lungs and are associated with higher clonality in tumor samples compared with never-smokers (person who has never smoked, or less than 100 cigarettes in their life) or long-term quitters.^28^^,^^32^ Clinical features and background immune states can therefore modulate the TCR signature independently of tumor characteristics, and these must be taken into consideration when interpreting repertoire-based biomarkers.

### Analysis in tumor samples (TILs)

**Figure 2:**
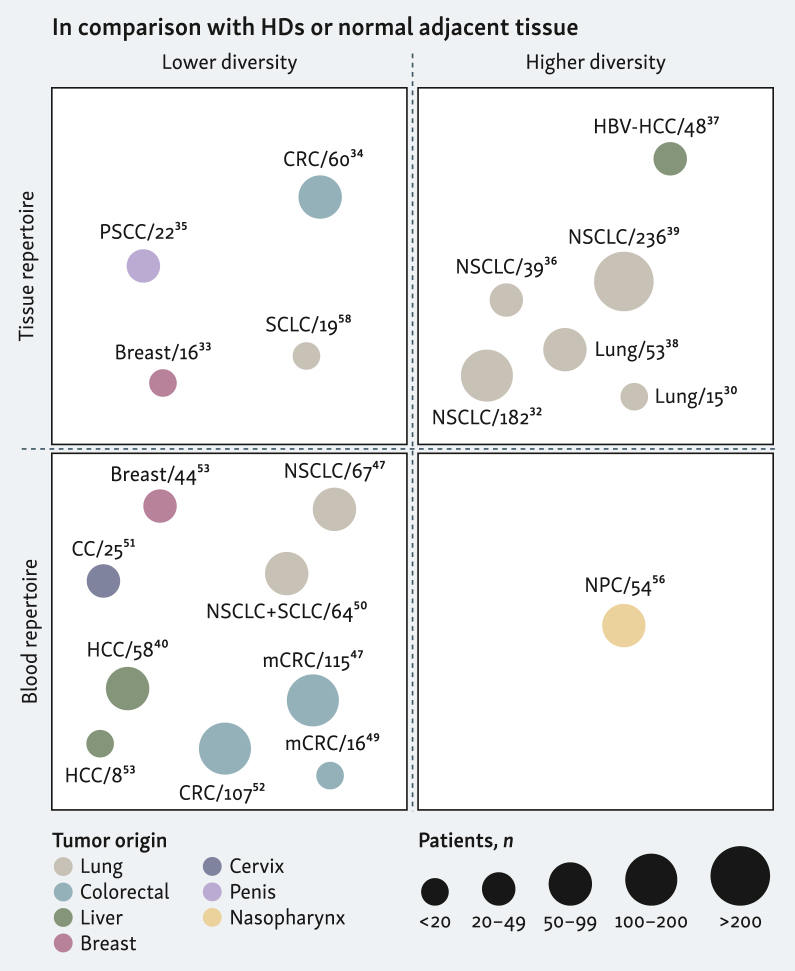
**Graphical representation of studies demonstrating changes in TCR repertoires associated with cancer status or diagnosis (related to**Supplementary Table S1**, available at**https://doi.org/10.1016/j.iotech.2025.101076**)**. Each quadrant displays TCR repertoire analysis in either tissue (top) or blood (bottom), contrasting low diversity (or high clonality, left) versus high diversity (or low clonality, right) when distinguishing cancer patients from healthy donors or malignant from paired normal tissues. Each circle indicates a different study; circle size reflects the number of patients in each study, and color corresponds to tumor type. Created with BioRender.com. CC, cervical cancer; HBV-HCC, hepatitis B virus–hepatocellular carcinoma; HD, healthy donor; (m)CRC, metastatic colorectal cancer; NPC, nasopharyngeal cancer; NSCLC, non-small-cell lung cancer; PSCC, penile squamous-cell carcinoma; SCLC, small-cell lung cancer.

TCR repertoire of tumor-infiltrating lymphocytes (TILs) is a promising tool for cancer diagnosis and staging. Some studies report that higher repertoire clonality (and by extension lower diversity) in tissue is often associated with malignant transformation (Figure 2).^33^, ^34^, ^35^ However, not all tumor types behave identically. In hepatitis B virus (HBV)-related hepatocellular carcinoma (HCC) and lung cancer, higher TCR diversity and lower oligoclonal dominance are sometimes found in tumor compared with adjacent nonmalignant tissue,^30^^,^^32^^,^^36^, ^37^, ^38^, ^39^ while others report no significant difference between tumor and paired normal tissue.^40^^,^^41^ Similarly, patterns of TCR diversity and clonality vary with tumor stage or size, with some cancers showing decreased diversity (i.e. increased clonality) with progression,^35^^,^^36^^,^^42^ while others show decreased TCR clonality and increased diversity with advancement or invasiveness.^34^^,^^38^^,^^43^^,^^44^ A study using machine learning leveraging TCR V-J usage and CDR3 motifs achieved nearly 98% accuracy in distinguishing primary from metastatic lesions and differentiating colorectal cancer (CRC) from gastric cancer, highlighting the diagnostic and prognostic value of TIL repertoire analysis.^44^ Benítez and colleagues further delineated molecular subtypes of muscle-invasive bladder carcinoma based on T-cell infiltration, clonal expansion and diversity^45^ and large-scale computational analyses in pediatric brain tumors demonstrated that TCR motif usage and diversity robustly differentiate primary from metastatic tumors.^46^ In summary, tumor-based TCR repertoire profiling can distinguish tumor from normal tissue and stratify cancer stage across various solid tumors (Supplementary Table S1, available at https://doi.org/10.1016/j.iotech.2025.101076 and Figure 2). However, diversity patterns vary by tumor type, underlying etiology, the tumor microenvironment and methodology (Supplementary Table S1, available at https://doi.org/10.1016/j.iotech.2025.101076), likely reflecting differences in T-cell infiltration and tumor mutational burden (TMB) between individuals and across paired tissues.^4^ Larger studies and comprehensive benchmarking are needed but current data highlight the strong potential of TCR profiling in different clinical settings.

### Analysis in peripheral blood mononuclear cells (PBMCs)

Several blood-based biomarkers are routinely explored for cancer diagnosis, including cell-free DNA (cfDNA), circulating tumor cells (CTCs) or circulating tumor DNA (ctDNA), and circulating microRNAs (miRNAs).^47^ However, each faces practical limitations: cfDNA may derive from nontumor sources, CTCs are scarce and short-lived, ctDNA is present at very low levels, and miRNA quantification remains technically demanding.^47^ Although these approaches have shown promise, these constraints complicate early and accurate cancer detection. In contrast, peripheral blood TCR repertoire profiling offers a minimally invasive and sensitive tool. Relative to TIL populations, characterized by expansion of putative tumor-reactive (or at times, bystander) clones, peripheral T cells exhibit a more diverse architecture.^31^^,^^40^^,^^48^ Numerous studies show that cancer patients exhibit altered blood TCR repertoires compared with healthy donors—typically with shifts in V/J gene usage and reduced diversity, often driven by decreased richness and the emergence of expanded clones, potentially reflecting tumor-reactive cells.^40^^,^^47^^,^^49^, ^50^, ^51^, ^52^, ^53^, ^54^, ^55^, ^56^ This may result from chronic antigen stimulation leading to clonal expansion, T-cell exhaustion and overall diversity loss. In some cases, like nasopharyngeal carcinoma, an opposite trend of increased TCR diversity in patients has been observed, possibly reflecting a broad but functionally impaired repertoire due to low TCR affinity, poor antigen presentation or T-cell exhaustion.^56^ Notably, Ji and colleagues demonstrated that circulating tumor-associated T cells, defined by shared TCR clones in blood and tumor, exhibit high clonality and tumor reactivity, and that combining blood TCR repertoire features with gene expression signatures significantly improves noninvasive cancer detection across multiple tumor types.^53^ These findings underscore the diagnostic value of blood-based TCR profiling for distinguishing cancer patients from healthy individuals, with patients generally showing lower blood TCR diversity, while data are more contrasted for tumor repertoires (Supplementary Table S1, available at https://doi.org/10.1016/j.iotech.2025.101076 and Figure 2). In peripheral repertoires, tumor-specific lymphocytes may be diluted and TCR diversity can be influenced by age, prior antigen exposure or immunosuppression. Analyzing TCRs at the tumor site may give more precise insights into the immune response, though studies comparing tumor and adjacent tissue repertoires show mixed results. Higher TCR diversity reflects a functional immune system capable of antitumor responses, whereas reduced diversity may indicate aggressive tumors or active antitumoral immunity. Moreover, machine learning frameworks leveraging blood TCR sequences can enable sensitive and specific cancer detection,^40^ even years before clinical diagnosis,^54^ especially when combined with other biomarkers like the gut microbiome in CRC or ctDNA and proteins in multi-analyte lung cancer screening.^52^^,^^55^ For instance, in nasopharyngeal carcinoma, a specific circulating TCR signature was shown to provide accurate early diagnosis outperforming standard serology diagnostic tests and to serve as a dynamic marker of disease onset, with higher signal intensity predicting a shorter time to clinical presentation.^57^ Statistical approaches can also distinguish early from advanced disease or metastatic risk,^48^^,^^50^^,^^51^^,^^58^ and repertoire similarities between blood and tumor can identify subtypes in lung cancer.^42^ Overall, blood TCR profiling is a promising minimally invasive tool for cancer detection, but data interpretations should account for disease type and other factors (Supplementary Table S1, available at https://doi.org/10.1016/j.iotech.2025.101076).

### Prognostic value in cancer patients treated or not with traditional treatments

After cancer diagnosis, TCR repertoire profiling emerges as a valuable prognostic biomarker, offering insight into antitumoral immunity and guiding therapeutic decision making. Assessing TCR diversity in patients, whether untreated or undergoing conventional cancer treatments (Supplementary Tables S1 and S2, respectively, available at https://doi.org/10.1016/j.iotech.2025.101076), helps predict disease progression and overall outcomes.

### Tumor and blood TCR repertoires as prognostic biomarkers of patient survival and disease-free survival

**Figure 3:**
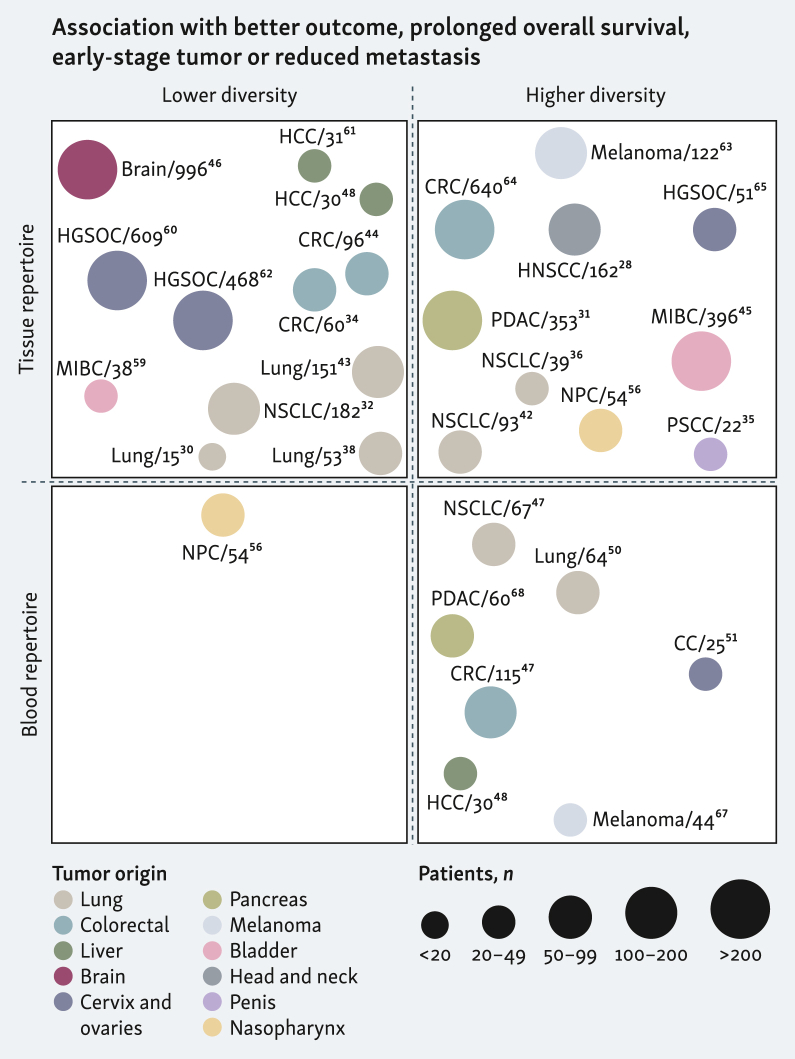
**Graphical representation of studies linking profiles of TCR repertoire with disease progression or patient survival (related to**Supplementary Table S1**, available at**https://doi.org/10.1016/j.iotech.2025.101076**).** Each quadrant displays TCR repertoire analysis in either tumor (top) or blood (bottom), comparing low diversity (or high clonality, left) and high diversity (or low clonality, right) in relation to favorable cancer outcomes such as good prognosis, improved overall survival, and less advanced disease or metastasis. Each circle indicates a recent study; circle size reflects cohort size, and color corresponds to tumor type. Created with BioRender.com. CC, cervical cancer; CRC, colorectal cancer; HCC, hepatocellular carcinoma; HG(S)OC, high-grade (serous) ovarian cancer; HNSCC, head and neck squamous-cell carcinoma; MIBC, muscle-invasive bladder cancer; NPC, nasopharyngeal carcinoma; NSCLC, non-small-cell lung cancer; PDAC, pancreatic ductal adenocarcinoma; PSCC, penile squamous-cell carcinoma.

Although controversial, a focused and oligoclonal repertoire of tumor-infiltrating T-cell, likely targeting tumor antigens, is frequently linked to improved survival across cancers—including bladder, lung, melanoma, ovarian, brain, and hepatocellular cancers (Figure 3).^30^^,^^32^^,^^46^^,^^48^^,^^59^, ^60^, ^61^ Strikingly, ovarian tumors with limited T-cell infiltration, yet focused repertoires, can match outcomes of highly infiltrated tumors, emphasizing TCR quality over quantity.^62^ In contrast, some other studies associated higher intratumoral TCR diversity (polyclonality) with improved disease-free survival and effective tumor control (Figure 3). For example, increased repertoire diversity correlated with improved survival in melanoma, nasopharynx cancer, microsatellite-stable CRC, high-grade serous ovarian cancer, pancreatic ductal adenocarcinoma, and certain bladder cancer subtypes, and it was hypothesized by these studies that a broad immune response enables durable tumor surveillance.^31^^,^^45^^,^^56^^,^^63^, ^64^, ^65^ Integrating TCR data with other immune and genomic signatures, such as T-cell infiltration, genomic alterations or gene expression, boosts prognostic accuracy and clinical relevance.^28^^,^^60^^,^^64^^,^^66^ In certain cancers, such as HBV-associated HCC and nasopharyngeal carcinoma, high overlap between tumor and adjacent tissue repertoires predicted better prognosis,^40^^,^^41^^,^^56^ though in non-small-cell lung cancer (NSCLC), a more tumor-focused landscape, reflected by low overlap with adjacent lung tissue, was beneficial.^39^ TCR profiling has also distinguished non-small-cell from small-cell lung cancer, the latter harboring a ‘cold’ TCR landscape, characterized by low richness and clonality, potentially explaining its notoriously poor prognosis.^58^ Nevertheless, because tumor sampling is invasive, the future of noninvasive immune monitoring and prognostication may hinge on identifying robust TCR-based biomarkers in peripheral blood.

As for blood-based TCR features, most studies converge to define repertoire richness as a good prognostic indicator. For instance, in melanoma, patients with a diverse repertoire of circulating T cells have prolonged progression-free survival and lower risk of relapse compared with those with restricted or oligoclonal blood repertoires.^67^ Similarly, in lung, CRC and pancreatic cancers, greater TCR richness in PBMCs reflects immune robustness and is associated with improved overall prognosis.^47^^,^^50^^,^^68^ In contrast with this apparent consensus, a study in nasopharyngeal carcinoma linked higher blood TCR diversity to poorer outcome, potentially reflecting the lack of dominant tumor-reactive clonotypes.^56^ Prognostic significance of blood TCR diversity can be decoupled from TIL features: in ovarian cancer, both the overlap and divergence between blood and tumor repertoires independently influenced clinical outcome, suggesting that systemic and tumor-local immune responses provide complementary information.^62^ High peripheral T-cell density also predicted better survival in NSCLC, reinforcing the value of systemic immune monitoring.^39^ Overall, higher peripheral blood TCR diversity reflects greater immune competence and predicts improved disease-free survival and better outcome, whereas conclusions regarding tumor repertoire diversity and its prognostic significance are more nuanced (Figure 3).

### TCR repertoire as a predictive biomarker of clinical benefit after conventional therapy

Emerging evidence indicates that TCR repertoire profiling can predict response and survival after conventional cancer treatments like surgery, chemotherapy and radiotherapy, which are increasingly recognized to involve immune-mediated mechanisms (Supplementary Table S2, available at https://doi.org/10.1016/j.iotech.2025.101076). As objective and noninvasive tools for monitoring therapeutic efficacy remain limited, blood-based TCR analysis stands out as a promising solution for preoperative risk assessment and response prediction. In human papilloma virus (HPV)16-driven cancers, increased blood clonality after chemoradiation predicted better prognosis, reflecting the expansion and maintenance of tumor-specific clones.^69^ Clonality alone does not always correlate with baseline diversity; rather, favorable outcomes are sometimes linked to the selective preservation and expansion of effective TCR clones, as seen in nasopharyngeal carcinoma, where nonmetastatic patients showed expansion of dominant clones with maintained diversity, while a drop in diversity with rising clonality identified poorer prognosis.^70^ Similarly, a longitudinal loss in blood TCR diversity during chemotherapy correlated with favorable responses in metastatic CRC.^49^ Importantly, in both studies (nasopharyngeal and CRC^,^^49^^,^^70^), sustained similarity between pre- and post-treatment TCR repertoires correlated with better outcomes, while greater repertoire changes were associated with improved recurrence-free survival in rectal cancer after chemoradiotherapy.^71^ In ovarian cancer, increased diversity of blood repertoire after poly (ADP-ribose) polymerase (PARP) inhibitors (after chemotherapy) was related to longer progression-free survival.^72^ Greater tumor-infiltrating TCR diversity also distinguished patients with fully resectable disease (and thus, a more favorable prognosis).^73^ Breast cancer studies are mixed, with some linking increased peripheral CD8+ T-cell diversity during chemotherapy with better treatment response,^74^ while a reduction in diversity predicted a more favorable response in others.^75^^,^^76^ Post-treatment blood TCR clonality is associated with improved survival, while baseline clonality combined with CTC counts can aid outcome prediction in metastatic breast cancer.^77^ In B-cell lymphoma, high tumor TCR clonality predicted inferior survival, and broader diversity marked better outcomes, with similar trends found in rectal cancer.^71^^,^^78^ Looking at blood repertoires, consistent observations indicated that higher peripheral diversity in CRCs and lung cancers predicted improved therapeutic response.^47^^,^^49^^,^^79^ Overall, TCR repertoire diversity (both at baseline and dynamic changes after therapy) in blood or tumor emerges as a robust predictor of conventional treatment outcomes and TCR profiling is foreseen as a source of potential actionable biomarkers in these clinical settings (Supplementary Table S2, available at https://doi.org/10.1016/j.iotech.2025.101076). Baseline TCR repertoire features are particularly valuable, since starting a patient on therapy merely to assess potential benefit is not practical.

## T-cell repertoire as a biomarker for immunotherapy outcomes

Immunotherapy has revolutionized cancer care by harnessing T cells to target malignancies through approaches encompassing adoptive cell transfer (ACT), vaccines and ICI therapies.^80^ Traditional biomarkers like programmed death-ligand 1 (PD-L1), mismatch repair deficiency and TMB require tumor tissue and do not fully capture the complexity of the host immune response.^5^ Their predictive value can also vary widely across cancer types. Consequently, there is an urgent need for minimally invasive, immune-centric biomarkers that reflect both the state and adaptability of a patient’s T-cell response. TCR repertoire sequencing could address this gap by enabling real-time, sensitive monitoring of T cells at the clonal level, providing powerful insights for predicting immunotherapy benefit and advancing personalized cancer care (Figure 1).^3^^,^^4^

### Immune profiling as predictive biomarker for ICIs

**Figure 4:**
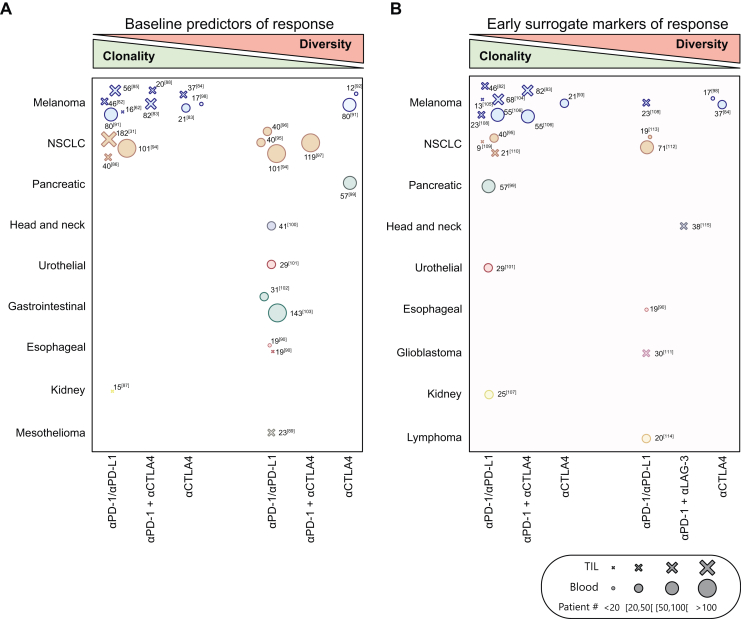
**Repertoire characteristics of responders to immune checkpoint inhibitors (ICIs).** Associations between repertoire clonality and diversity to ICIs treatment outcomes, either as baseline predictors of efficacy (A) or as early surrogate longitudinal markers of efficacy (B). Cancer types are listed according to their tumor mutational burden and each symbol corresponds to a distinct study. The type of ICI treatment appears at the bottom. Size of the dot, as well as the number, indicates the cohort size. (A) Studies are positioned based on the association between clinical benefit and either higher baseline repertoire clonality (left side) or higher baseline repertoire diversity (right side). (B) Studies are positioned based on the association between clinical benefit after ICI and changes in T-cell receptor repertoire (increased clonality or diversity on the left or right side, respectively). aCTLA-4, anti-cytotoxic T-lymphocyte-associated protein 4; aLAG-3, anti-lymphocyte-activation gene 3; aPD-1, anti-programmed cell death protein 1; aPD-L1, anti-programmed death-ligand 1; NSCLC, non-small-cell lung cancer; TIL, tumor-infiltrating lymphocyte.

Over the last decades, immunotherapy has emerged as a highly promising approach for cancer treatment. The approval of antibodies against cytotoxic T-lymphocyte-associated protein 4 (CTLA-4) and programmed cell death protein 1 (PD-1)/PD-L1 has significantly transformed the cancer treatment landscape, leading to a number of clinical trials assessing ICI efficacy.^4^^,^^80^ Despite their undeniable success, only a limited number of patients derive benefit from ICIs.^5^ Consequently, identifying reliable biomarkers to predict patient responsiveness has become a pressing need. Although the immunological mechanisms behind ICI treatment remains partially unclear between the reinvigoration of existing tumor-specific clonotypes versus the priming of *de novo* clonotypes,^81^ the central role of cellular immunity in the clinical benefit to immunotherapy is beyond doubt, indicating that granular TCR repertoire profiling holds the promise to both predict efficacy before treatment initiation or be used as a surrogate marker of clinical response to ICIs (Supplementary Tables S3 and S4, available at https://doi.org/10.1016/j.iotech.2025.101076 and Figures 4A and B, respectively).

Numerous studies have examined whether TCR repertoire metrics can help forecast patient outcomes. Researchers have explored TCR repertoires from TILs and peripheral blood, seeking associations between clinical benefit and repertoire diversity or clonality. Of note, while several correlates of efficacy were identified, no universal consensus was observed (Supplementary Table S3, available at https://doi.org/10.1016/j.iotech.2025.101076). Nevertheless, an overarching pattern appears to be emerging. When tumor samples were analyzed, several studies in melanoma and lung cancer and one study in kidney cancer have consistently linked higher TIL repertoire clonality with clinical benefit to anti-PD1/PD-L1 and/or CTLA-4;^32^^,^^62^^,^^82^, ^83^, ^84^, ^85^, ^86^, ^87^, ^88^ however, fewer and smaller studies in esophageal and mesothelioma reported the opposite trend (Figure 4A).^89^^,^^90^ As for studies investigating blood TCR repertoires, different observations in patients with melanoma or lung cancer reported contradicting findings, linking either higher clonality or higher diversity to clinical benefit (Figure 4A).^91^, ^92^, ^93^, ^94^, ^95^, ^96^, ^97^, ^98^ In contrast, in several other indications including pancreatic, head and neck, urothelial, gastrointestinal, or esophageal cancers, all studies (to the best of our knowledge) have observed an association between higher blood repertoire diversity and clinical benefit (Figure 4A).^90^^,^^99^, ^100^, ^101^, ^102^, ^103^ Of interest, in pancreatic adenocarcinoma, greater diversity in the peripheral blood TCR repertoire was associated with improved survival in patients receiving anti-CTLA-4 therapy, but not in those receiving anti-PD-1 therapy,^99^ consistently with the common hypothesis that CTLA-4 blockade mainly promotes naive T-cell priming while PD-(L)1 blockade acts on antigen-experienced T cells.^4^ Variability in findings can be attributed, at least in part, to factors such as the source of immune cells (e.g. peripheral blood versus TILs), the analytical methods and the specific metrics chosen. Additionally, individual patient history plays a significant role in shaping the antitumoral immune response and hence the outcome following therapies. Elements like age, cancer type and its associated TMB, prior surgical interventions, or chemotherapy can all influence immune function and, consequently, patients’ response to ICIs. To better understand the potential of TCR repertoire profiling as a biomarker, more rigorous studies with standardized methodologies and more homogeneous patient cohorts are essential. Encouragingly, the growing adoption of ICIs as a first-line therapy for cancer will make such studies increasingly feasible in the near future. Overall, TCR repertoire profiling shows promise as a predictive biomarker for ICIs, with higher TIL clonality or peripheral blood diversity often correlating with clinical benefit, though findings vary by cancer type, therapy, and patient factors. Standardized, rigorous studies are needed to clarify these associations and harness immune profiling for predicting ICI responses.

### T-cell repertoire for longitudinal response monitoring in ICIs

Beyond its predictive capabilities before immunotherapy, dynamic changes in TCR repertoires have also been explored as an on-treatment biomarker of ICI clinical efficacy (Supplementary Table S4, available at https://doi.org/10.1016/j.iotech.2025.101076 and Figure 4B). Similarly to the prediction of efficacy, which seems to depend on multiple factors such as TMB, cancer type and samples analyzed, the dynamic changes observed in TCR repertoires from patients receiving and benefiting from ICI are not universally consensual. Yet, there is a trend toward an increase in immune repertoire clonality (in both blood and tumor) in patients responding to ICI (Figure 4B).^82^^,^^83^^,^^93^^,^^95^^,^^99^^,^^101^^,^^104^, ^105^, ^106^, ^107^, ^108^, ^109^, ^110^ In melanoma, several studies consistently reported an increased clonality in blood or tumor from patients benefiting from anti-PD-1/anti-PD-L1 therapy, either alone or combined with anti-CTLA-4.^82^^,^^83^^,^^104^, ^105^, ^106^^,^^108^ Of interest, a study on 23 melanoma patients showed that responders to anti-PD-1 monotherapy displayed conjointly an increased clonality and richness (and by extension diversity) in their tumor upon treatment.^108^ Treatment with anti-CTLA-4 alone, in contrast, led to discordant observations.^84^^,^^93^^,^^98^ We report here three melanoma studies evaluating blood TCR repertoire shifts after anti-CTLA-4 therapy and their association with patient response. One of the studies found that increased peripheral clonality correlated with better patient outcomes,^93^ while the two others reported that enhanced diversity was more advantageous.^84^^,^^98^

Beyond melanoma, studies of TCR repertoire dynamics as early surrogate biomarkers of response to anti-PD-1 monotherapy have yielded contrasting results. Within tumors, PD-1 blockade was associated with increased clonality in lung cancers^99^^,^^110^ (as for melanoma), whereas greater diversity was linked to favorable outcomes in glioblastoma.^111^ In peripheral blood, increased repertoire clonality correlated with benefit in pancreatic, urothelial, kidney and lung cancers,^95^^,^^99^^,^^101^^,^^107^ with contrasting results in lung cancer where two other studies instead associated increased diversity with response.^112^^,^^113^ This was also supported with findings in esophageal cancer and lymphoma, where responders exhibited higher on-treatment diversity.^90^^,^^114^ Additionally, Li and colleagues demonstrated that combining anti-PD-1 with anti- lymphocyte-activation gene 3 (LAG-3) led to increased TCR diversity compared with the combination of anti-PD-1 and anti-CTLA4.^115^ These inconsistencies may be attributed to the variable timing of repertoire assessments, highlighting the importance of longitudinal studies to better elucidate TCR dynamics and improve predictions of treatment response. In addition to some controversial conclusions in certain tumor types, the perspective to turn the consensual observations based on baseline repertoire evaluation and longitudinal follow-up into actionable, clinically compatible biomarkers remains a challenge for multiple reasons. TCR repertoires, in particular in the blood, are dynamic by essence, indicating that TCR repertoire metrics will likely have to be normalized to enhance their prognostic value. Additionally, it has been shown that outcomes can vary depending on the repertoire analytical method used, as amplification-related biases can skew metric values and significantly impact patient stratification. Therefore, selecting the most reliable TCR sequencing technique would be crucial for accurately monitoring the repertoire and identifying the most suitable inhibitor. In conclusion, longitudinal monitoring of TCR repertoires during ICI therapy can serve as an on-treatment biomarker, with trends toward increased clonality or diversity often observed in responders, though results vary by cancer type, therapy and sampling method. Standardized longitudinal assessments and robust sequencing techniques are essential to reliably interpret TCR dynamics and translate them into clinically actionable biomarkers.

### Development and characterization of cell-based therapies

ACT therapies, encompassing traditional TIL infusions, genetically engineer TCR-T cells and chimeric antigen receptor (CAR)-T cells, leverage (ACT therapies leverage) tumor-reactive T cells for cancer treatment, but their efficacy is often limited by the scarcity of tumor-specific TCR clonotypes (or tumor targets) and poor persistence of the adoptively transferred clones *in vivo*.^116^ TCR repertoire analysis has emerged as a key tool for unraveling the mechanisms of action and monitoring efficacy in ACT therapies (Supplementary Table S5, available at https://doi.org/10.1016/j.iotech.2025.101076).^116^, ^117^, ^118^, ^119^, ^120^, ^121^, ^122^ By dissecting the clonal architecture of the therapeutic product, TCR sequencing helps distinguish genuine tumor-specific T cells from bystander cells, thereby guiding optimization of manufacturing protocols. Preclinical studies reveal that variables such as cytokine milieu and expansion methods critically shape the repertoire, sometimes favoring *in vitro*-fit bystanders over *bona fide* tumor-reactive clones.^123^, ^124^, ^125^ Clinically, analyzing infused products’ repertoires has demonstrated that enrichment for tumor-derived clonotypes—rather than those originating from peripheral blood—predicts better responses in melanoma (with enrichment for tumor reactivity)^116^ and that increased clonality of expanded CD8+PD1+ T cells correlates with improved outcomes in advanced gastric cancer.^119^

Beyond product analysis, baseline TCR repertoire profiling is emerging as a powerful predictor of patient benefit. Higher diversity and the presence of tumor-derived or neoantigen-specific clones in pretreated tumors are linked to improved clinical outcomes across ACT modalities, including TIL-ACT in melanoma and CAR-T in myeloma.^116^^,^^121^ After infusion, early tracking of TCR clonotypes in blood or tumor can signal success or relapse. In CAR-T therapy, initially polyclonal products often become dominated by a few clones after infusion.^118^ In affinity-enhanced TCR therapies for synovial sarcoma, durable responses reflect clonal persistence and regenerative memory T-cell pools,^120^ while in a patient with CRC who received an enriched product, durable tumor remission correlated with persistence, though tumor escape could occur via immune editing, including HLA loss.^117^ In melanoma and NSCLC, rapid loss of tumor-antigen-specific T-cell clonotypes after TIL therapy signaled poor response and immune evasion.^116^^,^^122^ Finally, in hematologic malignancy, remission after stem cell transplant was associated with a richer, more polyclonal antigen-specific TCR repertoire, highlighting the value of TCR sequencing for monitoring and risk stratification.^126^ Technological innovations, such as high-throughput TCR discovery (e.g. SEQTR),^7^ functional genetic screening,^127^ and integration with single-cell RNA-seq and tumor-reactivity gene signatures,^128^ facilitate the identification of predictive biomarkers and novel therapeutic TCRs, paving the way for next-generation personalized immunotherapies. Ultimately, TCR repertoire analysis—leveraging bulk, single-cell and functional approaches—has become essential for optimizing, tracking and personalizing cell-based cancer therapies. It is thus likely that longitudinal blood monitoring of immune repertoire after ACT may become a routine companion diagnostic in ACT therapies.

### Cancer vaccines

Dr Coley, known as the father of cancer immunotherapy, pioneered the concept of cancer vaccines two centuries ago by injecting microbial agents to treat cancer. The recent explosion of knowledge enabling the identification of private tumor neoantigens has created a second momentum in the field and opened the door to personalized cancer vaccines.^129^ Since vaccine-induced immune activation is expected to lead to proliferation of antigen-specific T cells, longitudinal analyses of TCR repertoires have been instrumental to tracking clonal dynamics and evaluating treatment responsiveness (Supplementary Table S5, available at https://doi.org/10.1016/j.iotech.2025.101076). Multiple studies in various cancer types have reported that therapeutic vaccination is associated with the expansion of T-cell populations, with specific clonotypes identified as reactive to tumor antigens.^130^, ^131^, ^132^, ^133^, ^134^ Moreover, evidence from Hsu and colleagues suggests that an increased overlap of the TCR repertoire between TILs and peripheral blood correlates with improved clinical outcomes.^135^

Furthermore, emerging evidence indicates that preventive vaccines, beyond those targeting infectious agents such as papillomavirus for cervical cancer, are in reach.^136^ Collectively, these findings support the utility of TCR repertoire profiling as valuable for monitoring the immunogenicity and predicting the efficacy of therapeutic and prophylactic cancer vaccination strategies.

## Discussion and perspective

The past decade has seen remarkable progress in leveraging TCR repertoire analysis for cancer immunology, with high-throughput sequencing unlocking new opportunities for diagnosis, prognosis and treatment monitoring. Blood-based TCR profiling, in particular, has emerged as a promising, minimally invasive tool for risk stratification, real-time immune surveillance and personalized cancer care. Studies consistently demonstrate that reduced peripheral TCR diversity distinguishes cancer patients from healthy controls across tumor types. While an oligoclonal or more focused intratumoral TCR repertoire is frequently associated with better outcomes, peripheral TCR diversity more often reflects improved immune competence and survival. As ICIs and other novel immunotherapies reshape the treatment landscape, dynamic and baseline TCR repertoire features are emerging as valuable, though complex, biomarkers for response prediction and disease monitoring.

However, despite the promise, significant limitations temper the immediate clinical translation of TCR repertoire analysis. The literature is marked by heterogeneity in patient cohorts, tumor types, prior treatments and sampling (primary versus metastatic lesions), all of which confound direct comparison and generalization. Particularly, in immune-desert tumors, TCR sequencing is limited by the paucity of tumor-infiltrating T cells, so most signals may derive from bystander, tissue-resident or perfusing peripheral T cells; thus, a quantitatively (low density) and qualitatively (low richness and clonality) cold TCR repertoire means no robust antitumor signature can be inferred. Interpretation of TCRseq in these scenarios must account for low T-cell density and diversity, preventing strong diagnostic or predictive conclusions. Technical challenges—including differences in sample preparation, sequencing technology, metric standardization and computational pipelines—further complicate cross-study interpretation. The use of global repertoire metrics such as diversity and clonality alone is insufficient to capture the full complexity of antitumor immunity, and *in silico* clustering and motif analyses, while promising, remain largely exploratory. Additionally, the immune contexture is influenced by factors such as infection, inflammation and individual genetics, limiting biomarker specificity. Most studies are retrospective, limited in size and lack prospective validation or unified analytic standards, highlighting the need for harmonized clinical trials and standardized methodologies.

Addressing these limitations is critical for moving the field forward. The contradictory findings on whether high diversity or high clonality predicts response are likely rooted in specific biological and therapeutic contexts. For instance, the mechanism of the immunotherapy itself is a key variable: anti-CTLA-4 therapy, which acts early to broaden the T-cell response, may favor patients with a higher baseline peripheral diversity capable of generating *de novo* responses. In contrast, anti-PD-1/PD-L1 therapy, which primarily reinvigorates existing exhausted T cells within the tumor, may be more effective in patients who already have a focused, highly clonal intratumoral response. Furthermore, the timing of the analysis is paramount; a pretreatment snapshot captures immune potential, while an on-treatment analysis reflects the dynamic clonal expansion or contraction that defines the therapeutic response. The contradictory findings in different tumor indications are also raising questions as to whether they are rooted in study-specific differences or rather reflect inherent distinct patterns of antitumoral T-cell responses in different cancer indications. Future studies must therefore stratify analyses not just by cancer type, but also by the specific therapeutic agent, the timing of sample collection and underlying tumor characteristics like TMB and the ‘hot’ versus ‘cold’ nature of the microenvironment.

Looking ahead, integrating TCR sequencing with single-cell technologies, advanced analytics and multi-omic approaches will move the field beyond descriptive immunogenomics toward actionable, precision immunotherapy. In the future, granular TCR repertoire profiling could be central to cancer diagnosis, patient stratification, early detection of immune toxicities and personalized treatment adaptation. Beyond oncology, TCR repertoire analysis is emerging as a key tool in transplantation (for monitoring rejection), autoimmunity (for disease characterization and monitoring) and infectious diseases (for tracking adaptive immune responses). As these applications expand, TCR profiling is set to become a cornerstone of precision medicine across a range of immunological conditions, ushering in a new era of immune-driven precision medicine. Integrating TCR sequencing with other patient-specific immune and clinical data could provide a personalized view of antitumor T-cell potency, akin to a modern ‘cancer immunogram’.^137^ This would require longitudinal datasets and computational approaches to synthesize diverse data into coherent, patient-specific profiles, potentially informing precision immunotherapy.

Realizing this vision requires more than resolving scientific complexities; significant practical hurdles must also be overcome for TCR profiling to become a routine clinical biomarker. The transition from a research tool to a validated clinical diagnostic test involves more than reducing sequencing costs. Firstly, there is a need for rapid turnaround times—clinicians require actionable data within days, not weeks, to guide treatment decisions. Secondly, complex bioinformatic analysis requires specialized expertise that is not standard in most clinical laboratories. Standardized, automated pipelines with clear, interpretable reports are essential. Finally, and most importantly, prospective clinical trials must be designed to demonstrate not just correlation but clinical utility. A TCR-based biomarker must be clearly linked to a specific clinical action, for example, guiding the choice between monotherapy and combination immunotherapy, or identifying patients who would benefit from adoptive cell therapy. Without this proven actionability, even the most robust predictive biomarker will remain a tool for research rather than a cornerstone of personalized medicine.

## Declaration of generative AI and AI-assisted technologies in the writing process

During the preparation of this work the authors used Anara (GPT 4.1) and ChatGPT in order to improve readability and language. After using this tool/service, the authors reviewed and edited the content as needed and take full responsibility for the content of the publication.
