# Supplementary material for: Harnessing TCR repertoires: predictive insights and therapeutic monitoring in cancer immunotherapy
Source: Immunooncol Technol. 2025 Oct 1;28:101076. doi: 10.1016/j.iotech.2025.101076 (PMC12615767; doi:10.1016/j.iotech.2025.101076)
Supplement: Supplementary Table S1 [file mmc1.pdf]

Table S1 - Overview of studies linking TCR repertoire profiling to cancer diagnosis and patient survival

Cancer	Cancer type / patient	Sample	TCR-Seq method	TCR repertoire characteristics	TCR repertoire analysis	Association with patient diagnostic/survival	Publication year	Journal	Reference
Lung	Lung cancer / 15	Paired tumor and adjacent normal lung tissue	Nested PCR (primers according to protocols modified from ARM-PCR)	Richness, diversity (Simpson's and Shannon's entropy), HECs (highly expanded clones), V/J gene usage	Non-tumor lung had a higher frequency of highly expanded clones (greater clonal dominance, lower overall diversity). TRBV20-1 and TRBV18 segments more frequently used in tumor tissues than normal lung. Diversity decreased with patient age.	Tumors had significantly more unique TCR clones (higher richness) and higher overall TCR clonal diversity than matched normal lung tissue. Higher TCR diversity in tumor tissue was associated with worse outcomes --> may reflect tumor-driven ineffective or suppressed immune responses rather than robust anti-tumor activity.	2019	Genomics, Proteomics, Bioinformatics	Wang *et al.* [30]
	Advanced lung cancer (NSCLC & SCLC) / 64 + 31 HDs	Peripheral blood at baseline + after treatment for 26 patients	Multiplex PCR (in-house primers panel)	Richness, diversity (normalized Shannon's entropy), repertoire overlap (Morisita-Horn index) and V/J segment usage	-	Baseline peripheral TCR diversity was lower in patients than in HDs and diversity in patients with a more severe disease state or poorer immune status was substantially lower --> limited peripheral TCR repertoire diversity is worse for survival.	2019	International Journal of Cancer	Liu *et al.* [50]
	NSCLC / 236 + 11 COPD patients and 24 HDs	Baseline tumor tissue, adjacent normal lung tissue, and peripheral blood	ImmunoSEQ	Richness, clonality (1 - Pielou's evenness), repertoire sharing/similarity between tumor, adjacent normal lung, and blood (homology measures, shared/unique clone)	Substantial overlap existed between tumor and adjacent lung TCR repertoires, which may reflect shared mutational or viral antigens. Significantly higher clonality in uninvolved tumor-adjacent lung tissue compared to that of COPD patients and healthy lung donors. Tumor clonality correlates with presence of cytotoxic CD8+ T cells, markers of immune activation, and anti-tumor cytolytic potential.	T cell richness was significantly higher in the tumor compared to the uninvolved tumor-adjacent lung, while T cell clonality was highest in the uninvolved tumor-adjacent lung --> could reflect bystander T cell reactivity in the adjacent uninvolved lungs or an accumulation of exhausted tumor-reactive T cells outside the tumor microenvironment. A more tumor-focused repertoire (less overlap with adjacent lung tissue) is associated with better overall survival. They observed an association between higher T cell density in the blood and improved outcome following surgery.	2020	Nature Communications	Reuben *et al.* [39]
	NSCLC / 39	Paired tumor and adjacent normal lung tissue	Multiplex PCR (gDNA, in-house panel of 51 V-forward and 14 J-reverse primers)	Richness, diversity (Shannon-Wiener index), Highly Expanded Clones (HEC) ratio	-	Tumor and normal tissue samples displayed significantly distinct TCR repertoires (higher richness and lower HEC ratio in tumor). Reduction of TCR richness with disease progression. Developed a classifier model based on selected TCRβ V-J pair usage distinguishing patients at risk of postoperative recurrence.	2020	Molecular Therapy: Methods & Clinical Development	Song *et al.* [36]
	SCLC / 19	Paired tumor and adjacent normal lung tissue	ImmunoSEQ	Richness, density (fraction of DNA derived from T cells), clonality (1 - Pielou's evenness)	-	SCLC display a "cold" TCR landscape: low density, low richness and clonality (indicating restricted diversity) compared to adjacent normal lung tissue and to NSCLC tissue (from a different cohort). This "cold" TCR features may underlie the extremely poor outcome and lack of immunogenicity typical of this tumor type.	2021	Nature Communications	Chen *et al.* [58]
	Lung ADC (different stages) / 53	Paired tumor and adjacent normal lung tissue	ImmunoSEQ	T cell density (normalizing TCR-β template counts to the total amount of DNA usable for TCR sequencing), diversity (inverse Simpson index), clonality (1 - Pielou's evenness), T-cell infiltration and polarization (RNA-Seq data)	Progressive decrease in anti-tumor immunity and increase in immunosuppressive features parallels disease progression.	T cell clonality and frequency of top clones decrease with neoplastic progression, despite increased T cell density and diversity (suggesting more but less effective, more bystander T cell infiltration). Combining immune gene/protein, TCR repertoire, and genomic/epigenomic features may enhance patient stratification and biomarker development for early lung ADC. Anti-tumor immunity evolved as a continuum from lung preneoplasia, to preinvasive ADC, minimally-invasive ADC and frankly invasive lung ADC with a gradually less effective and more intensively regulated immune response including down-regulation of immune-activation pathways, up-regulation of immunosuppressive pathways, lower infiltration of cytotoxic T cells (CTLs) and anti-tumor helper T cells (Th), higher infiltration of regulatory T cells (Tregs), decreased T cell clonality, and lower frequencies of top T cell clones in later-stages.	2021	Nature Communications	Dejima *et al.* [38]
	NSCLC / 93	Baseline tumor tissue and postoperative peripheral blood	Multiplex PCR (in-house primers panel)	Diversity (Shannon index), clonality, similarity metrics (overlap, Morisita and Jaccard index) with mutational status (EGFR, TMB) determined by next-generation sequencing	Highest clonality and lowest Shannon index in tumors of patients with EGFR non-L858R mutations. Repertoire similarity between tissue and blood was lower in EGFR L858R and higher in non-L858R group.	Clonality was significantly higher in late stages vs early stages of lung cancer, while diversity was generally lower in advanced disease and in EGFR non-L858R mutation subtype. Higher clonality and specific similarity metrics (OLI, MOI) are associated with EGFR subtype, higher TMB, and lower CD28+ T-cell ratios - factors linked to different immunotherapy responses	2021	Frontiers in Oncology	Yang *et al.* [42]
	Multiple primary lung cancer (MPCL) / 27 vs solitary lung cancer nodule (SN) / 124	Baseline tumor tissue	Multiplex PCR (in-house primers panel)	Diversity (Shannon index), clonality, frequency of the top 100 clones, repertoire overlap (Morisita's index)	Significant clonal expansion of clones shared among all lesions of the same MPLC patient, indicating immune repertoire commonality within each patient. Considerable heterogeneity in TCR repertoire exists among not only different patients but also among different lesions of the same patient.	Distinct TCR expansion and mutation profiles can help differentiate MPLC from solitary nodules/metastases --> Invasive lesions of MPLC exhibited significantly higher TCR diversity and lower TCR expansion than those of SN. Shared TCR clonotypes could be harnessed as biomarkers for open, multi-lesion-targeted immunotherapy or for post-surgical monitoring in MPLC.	2024	Cancer Immunology, Immunotherapy	Wang *et al.* [43]
	NSCLC / 182	Baseline tumor tissue	TCR data inferred from RNASeq data	Richness and clonality (Gini index)	High clonality correlated strongly with distinct mutations (EGFR, P53), TMB, and inflamed tumor phenotypes with exhaustion signatures. The analysis of the 182 NSCLC cases revealed a heterogeneous distribution of clonality in samples, in median with lower Gini indexes (high diversity), with some few exceptions. None of the evaluated parameters (patient age, stage, and sex) correlated with the Gini index, with the only exception that ever-smokers revealed a higher TCR receptor clonality in their tumors compared to never-smokers.	TCR clonality in cancer tissue was lower than in matched normal lung. The survival analysis of the Gini index did not show a significant prognostic impact, but when applying an optimal cut-off (without using data censoring at 5 years), they observed a significantly increased survival of patients with higher Gini index, i.e. with dominant clones.	2025	BioRxiv	Yu *et al.* [32]
	Lung cancer / 463 + 587 HDs	Baseline peripheral blood	Multiplex PCR (from DNA, with specific primers)	Clustering into TCR repertoire functional units (RFUs) using sequence similarity	-	Built a diagnostic model based on ML, which performance was compared to circulating tumor DNA (ctDNA) mutation analysis and protein biomarker panels. The study identified 327 cancer-associated TCR RFUs, many of which correlated with patient HLA genotype and known tumor-infiltrating lymphocyte antigen reactivities. The ML model based solely on blood TCR RFU features detected nearly half of stage I lung cancers at 80% specificity, and its performance improved further when combined with ctDNA and circulating protein markers, boosting early cancer detection sensitivity by up to 20 percentage points over established analytes alone.	2025	NPJ Precision Oncology	Li *et al.* [55]
	MSS-CRC / 640 (582 confirmed MSS)	Baseline tumor tissue (surgery)	ImmunoSEQ	T-cell abundance (estimated as the normalized # of TCR reads over the estimate of the total # of cells), clonality (Simpson clonality index = square root of the Simpson diversity index)	TCR variables are independent of traditional factors (age, sex, tumor site, stage IIA/IIB). Lymphocyte activation or TCR signaling pathways correlated with TCR abundance.	Higher TCR abundance is strongly and independently linked to improved DFS, while higher clonality index (i.e., lower repertoire diversity) is associated with poorer DFS --> patients with both high TCR abundance and low clonality had the best prognosis.	2020	PLoS Medicine	Sanz-Pamplona *et al.* [64]
	Metastatic CRC (mCRC) / 16 + 20 HDs	Baseline peripheral blood	Multiplex PCR (iRepertoire)	Richness, diversity (Chao1 and Shannon's entropy) and specific clonotypes distribution	-	mCRC patients had altered (difference in V/J segments) and less diverse TCR repertoires compared to HDs. The lymphocyte to monocyte ratio may serve as a prognostic marker in colon cancer.	2021	Frontiers in Immunology	Chen *et al.* [49]
	CRC / 107 + 30 HDs	Peripheral blood	Multiplex PCR (from DNA, with specific primers)	Richness, diversity (Simpson index, Shannon-Wiener index), HECs, V/J gene usage,	The gut microbiome in patients with CRC significantly differed from that in HDs. CRC-implicated somatic mutations are related to TCR and the gut microbiome.	CRC patients showed significantly reduced TCR diversity compared to HDs, with more large, expanded TCR clones --> a random forest classifier integrating TCR clonotypes and microbiome markers achieved extremely high diagnostic accuracy: AUCs of 98.99% (early CRC) and 99.22% (late CRC) for distinguishing CRC from healthy individuals.	2023	Gut Microbes	Cao *et al.* [52]

Colorectal	CRC / 60 (40 with LN metastasis, 20 without)	Baseline tumor tissue (primary and metastatic LNs)	Multiplex PCR (RNA, Immune Repertoire Library Preparation Kit (Geneway, Jinan, China))	Diversity (UT and Shannon index), motif analysis and V/J gene usage	TCR repertoire is altered in CRC patients exhibiting LN metastasis, potentially influencing disease progression.	Immune repertoire diversity was lower in CRC patients than in healthy samples. Significant increase in richness and diversity (but lower UT index) in patients with LN metastasis compared to controls. Certain V-J combinations and a cohort of CDR3 motifs were specific to the LN positive group --> biomarkers for metastatic potential.	2024	Frontiers in Oncology	Zhen *et al.* [34]
	CRC / 96, GC / 47	Baseline tumor tissue (primary and metastatic)	Multiplex PCR (RNA, Immune Repertoire Library Preparation Kit (Geneway, Jinan, China))	Richness, diversity (Shannon and Simpson index, D50), clonality, motif analysis and V/J gene usage	CRC enriched for V/J combinations, while GC demonstrated higher gdT-cell-related recombination, as well as distinct amino acid preference between CRC vs. GC.	CRC showed later-stage increases in clonal diversity and distinct V-J/CDR3 characteristics with advancing stage. The immune repertoire in CRC metastatic lesions was more complex and diverse than in primary lesions. Multi-layer machine learning models achieved high accuracy distinguishing CRC from GC.	2025	Frontiers in Immunology	Yuan *et al.* [44]
	CRC / 115, NSCLC / 67 + 114 HDs and 56 controls with benign disease	Baseline peripheral blood	Multiplex PCR (from RNA, with specific primers)	Diversity (D50 value, Shannon entropy and Simpson index)	Lower D50 with advanced NSCLC stage (lymph node metastasis), and higher D50 correlated with normal carcinoembryonic antigen (CEA) levels in both cancers.	Lower diversity in CRC and NSCLC patients compared to HD, whereas begnin controls showed a modest decrease in TCR diversity. Higher D50 values correlated with normal CEA (a favorable prognostic marker) in both CRC and NSCLC. They identified various TRBV and TRBJ genes expressed differently among HDs and patients with CRC, NSCLC. In CRC, D50 and Shannon entropy were significantly higher in patients with better tumor regression grades.	2025	Cancer Medicine	Ma *et al.* [47]
Liver	HBV-HCC / 48	Paired tumor and adjacent normal liver tissue	5' RACE (SMARTer kit)	Richness, diversity (Shannon's entropy, Simpson index), clonal dominance (Gini index), HECs, V/J gene usage, clonal overlap (Morisita-Horn similarity index)	The usage patterns of Vβ and Jβ gene segments were similar between tumors and adjacent non-tumor tissues with some specific combinations appearing only in one tissue or the other (but at low frequencies), resulting in a weak similarity and overlap between tissues.	Tumor tissues display higher TCR repertoire diversity and lower oligoclonal dominance compared to adjacent tissue.	2016	Oncoimmunology	Chen *et al.* [37]
	HBV-HCC / 23	Paired tumor and adjacent normal liver tissue	5' RACE (SMARTer kit)	Richness, diversity (Shannon's entropy), clonality index ((1-Shannon's entropy)/log2(richness)), repertoire similarity (Morisita-Horn index)	TCR repertoire diversity between tumor tissues and matched adjacent normal tissues is not statistically different.	The combination of TCR repertoire similarity and TNM stage is better prognostic indicator for HCC patients (vs single marker) --> higher similarity of TCR repertoire between paired tissues was associated with better prognosis.	2018	Cancer Medicine	Lin *et al.* [41]
	HCC / 58	Tumor tissue, peritumoral tissue, and peripheral blood at baseline	For patients, multiplex PCR (using in-house primers) and for HDs, TCR data inferred from RNASeq from TCGA	Richness, diversity (Shannon's entropy), V/J gene usage, clonal overlap	Significantly higher TCR diversity in peripheral blood than in tumoral and peritumoral tissues, while tumoral and peritumoral tissues showed similar TCR diversity.	In blood: significantly higher diversity in healthy controls; unique VJ usage profiles distinguished HCC from healthy individuals --> peripheral blood TCR VJ pairing profiles are highly discriminative for HCC diagnosis.<br>In patients with microvascular invasion (MVI, a poor prognostic factor), there were fewer shared TCR clones between tumor and peritumoral tissues.	2022	Frontiers in Immunology	Wang *et al.* [40]
	HCC / 30	Paired tumor and adjacent normal liver tissue and peripheral blood at baseline	5' RACE amplification	Diversity (D75, Shannon, Hill, Gini-Simpson, inverse Simpson, Singleton indices), clonality (top 100 CDR3 fraction)	TCR diversity was consistently higher in PBMCs than tumor or adjacent tissues across all tumor stages and recurrence status.	PBMC diversity was a sensitive marker distinguishing disease burden/stage, showing marked reduction in advanced HCC; high clonality was a distinguishing feature of PBMCs in advanced disease. High clonal expansion of intratumoral T cells in early-stage HCC clearly correlated with lower risk of recurrence.	2023	Cancer Science	Li *et al.* [48]
	HCC / 31	Baseline tumor tissue	Multiplex PCR (RNA, Immune Repertoire Library Preparation Kit (Geneway, Jinan, China))	Diversity (Shannon and Simpson indexes), clonal expansion, V/J gene usage	Candidate TCR segments and CDR3 motifs from long-survivors may serve as molecular targets for antigen-specific immunotherapy development in HCC.	Lower TCR diversity and specific V/J segment usage were strongly associated with longer survival post-resection. Random forest classifier using TCR parameters robustly separated long and short-survivors.	2025	Technology in cancer research and treatment	Wang *et al.* [61]
Liver, lung, gastric, colonrectal, esophageal and breast	Multiple cancer types including liver, lung, gastric, CRC and esophageal cancers Validation cohort 1: breast cancer / 33, HCC / 8 and HDs / 12 Validation cohort 2: breast cancer / 11, HDs / 6	Baseline tumor tissue and peripheral blood	Different for each publically available cohort used / for their own validation cohort, multiplex PCR (ImmuHub TCR profiling)	Richness, clonality, Gini coefficient, repertoires overlap (defined TATs as tumor-associated T-cells - all circulating T lymphocytes clones in PBMCs also found within TILs)	TILs exhibited a higher fraction of TCRs shared with PBMCs ("shared" compartment) than PBMCs did with TILs. This difference can be interpreted as TILs being more enriched with circulating TATs. he shared compartments (clones found in PBMCs and TILs) exhibited specific TRBV gene usage, more frequently than in non-shared compartments.	Cancer patients' PBMCs had significantly higher TCR clonality and Gini coefficient than healthy individuals. TATs showed higher clonality and Gini coefficients (greater clonal expansion and inequality in clone distribution) than either PBMCs_Only or TILs_Only or healthy PBMCs. The combined TCR repertoire risk score and TAT signature gene score (transcriptomic markers in PBMCs) markedly improved the accuracy of non-invasive cancer detection, outperforming either metric alone for tumor screening.	2022	Clinical and Translational Medicine	Ji *et al.* [53]
Ovarian	HGOC / 369 from TCGA + 99 ovarian cancer patients as validation cohort	Tumor tissue (from both cohorts) and peripheral blood (validation cohort only), at diagnosis or during surgery (pre-treatment)	TCR data inferred from RNASeq from TCGA / ImmunoSEQ for validation cohort	Diversity (calculated as clontypes per kiloreads for inferred data or Shannon's entropy), clonality (1-Pielou's index), overlap & similarity between repertoires	Diverse tumor repertoires were matched by a similarly unfocused (=diverse) repertoire in the periphery.	Monoclonal TCR tumor repertoires (high clonality, low diversity) were associated with significantly better PFS and OS. Patients with low T cell infiltration but focused/monoclonal repertoires had outcomes comparable to highly-infiltrated tumors. High divergence between tumor and blood TCR repertoires (unique tumor clones) was associated with improved clinical outcomes.	2020	Oncotarget	Tsuji *et al.* [62]
	HGSOC / 578 from TCGA + 31 patients as validation cohort	Tumor tissue at diagnostic (pre-treatment)	TCR data inferred from TCGA WES	Clonality, abundance/infiltration --> data inferred from WES analysis	-	High TCR clonality is strongly associated with improved OS in HGSOC patients. Combining TCR clonality with T cell infiltration, HRD, BRCA, or CNV signatures improved prognostic performance versus any single marker.	2021	Cancers	Lecuelle *et al.* [60]
	HGSOC / 51	Baseline tumor tissue (surgery)	TCR data inferred from RNASeq downloaded from GEO database	Richness, diversity (Chao1 and D50 diversity index), expansion of specific V/J segments	-	Lower tumor TCR repertoire diversity was associated with recurrence, and recurrent cases often exhibited expansion of specific TCRs. Some genes co-expressed with TCRs differed significantly by prognosis --> gene expression linked to TCR metrics further refines risk stratification.	2023	Genes	Kim *et al.* [65]
	HGOC / 466	Peripheral blood (preoperative) and longitudinal pre-diagnostic peripheral blood (up to 5 years before diagnosis)	ImmunoSEQ	Richness, clonality/cluster structure (not directly calculated as Shannon entropy, but instead frequency and distribution in functional clusters), repertoire similarity and antigen specificity by sequence motif analysis (logo plots) --> GIANA: in-house embedding for TCR clustering	Transient but marked immune responses are reflected in TCR repertoire, peaking 4–2 years before diagnosis and decreasing as the tumor becomes immunosuppressive.	Strong, quantifiable changes in the TCR repertoire (OV RFU score) can be detected in blood up to 4 years before conventional diagnosis of HGOC.	2024	Cell Report Medicine	Yu *et al.* [54]
Bladder	MIBC / 38	Baseline tumor tissue	5' RACE (Clontech) followed by Ion Torrent next generation sequencer PGM	Richness and diversity (Simpson index)	Tumors with low diversity and low predicted neoantigen load showed higher expression of immunosuppressive genes (IDO1, FOXP3) and lower cytolytic gene expression (CD8, GZMA).	Lower TCR diversity in tumor tissue was associated with significantly longer RFS --> TCR repertoire diversity in bladder tumor tissue is an independent biomarker for recurrence risk post-cystectomy.	2016	European Urology Focus	Choudhury *et al.* [59]
	MIBC / 396	Baseline tumor tissue	TCR data inferred from RNASeq for TCGA	Richness, diversity (Shannon's entropy), clonal expansion (Gini index),	Comparison of MIBC subtypes: stroma-rich and basal/squamous-like (Ba/Sq) subtypes showed the highest TCR and BCR infiltration and clonal expansion; luminal papillary (LumP) had the lowest infiltration and diversity. In Ba/Sq, immune metrics correlated with inflammatory score, while in Stroma-rich, they correlated with TMB.	Higher TCR richness and diversity were significantly associated with improved overall survival specifically in Stroma-rich and Ba/Sq subtypes (not in Luminal subtypes).	2023	Frontiers in Immunology	Benitez *et al.* [45]
Pancreatic	PDAC / 1) 10 patients + 10 HDs, 2) 50 patients + 50 HDs	Baseline peripheral blood	scTCR-Seq (10x Genomics)	Richness, diversity (Shannon-Weiner and Inverse Simpson index), clonal overlap	-	Single-cell TCR repertoire profiling in blood reveals decreased overall T cell proportion and clonal diversity but expanded cytotoxic CD8+ T cells in PDAC. Reduced TCR clonal diversity, despite expansion of cytotoxic CD8+ T cells, was observed in PDAC and may contribute to poor outcomes.	2023	Frontiers in Endocrinology	Pan *et al.* [68]

Pancreatic	PDAC / 353	Baseline tumor tissue and peripheral blood	ImmunoSEQ	Diversity (Shannon Wiener, Inverse Simpson and "true entropy" indices) and specificity clustering	TCR diversity is increased in blood compared to tumor. TCR repertoire diversity (in PBMC and tumor) decreases with age but is not affected by neoadjuvant therapy, stage, or grade.	High intratumoral TCR diversity predicted better OS in PDAC cohorts. High T-cell abundance (not diversity) in blood predicted OS. A subset of TCRs ("metaclusters") were independently predictive of OS.	2024	Oncoimmunology	Pothuri *et al.* [31]
Skin	Melanoma / 44	Peripheral blood + metastatic LNs for 7 patients	Multiplex PCR (ImmunTraCkeR test)	Richness and diversity (ratio of the # of rearrangments necessary to reach 50% of frequency of the total repertoire / total # of rearrangments = richness)	-	Higher TCR repertoire diversity in blood was associated with longer PFS while low diversity was associated with rapid progression. Higher diversity at the tumor site (LNs) suggested possible additional prognostic value for intra-tumoral TCR analysis.	2020	Pigment Cell and Melanoma Research	Charles *et al.* [67]
	Melanoma / 122 TCGA cohort: 412 melanoma + others spanning >29 tumor types	Baseline (pre-treatment) tumor biopsies	RNA-seq data with computational TCR reconstruction (for pan-cancer public data)	Richness, diversity (Renyi index) and clonality (1–normalized Shannon entropy), repertoire similarity (Morisita–Horn and Simpson) and CDR3 motif analysis	-	High TCR diversity in pre-treatment tumor was strongly prognostic for improved overall survival in melanoma patients who did not receive immunotherapy, the same was also observed in several other tumor types (breast, renal, lung, thymoma, etc.). TCR clonality was not associated with prognosis in the absence of immunotherapy, reinforcing diversity as the relevant diagnostic/prognostic marker.	2021	Nature Communications	Valpione *et al.* [63]
Breast	Early stage breast cancer / 16	Paired tumor and adjacent normal breast tisue and peripheral blood	ImmunoSEQ	Richness, clonality (1 - Pielou's evenness), T-cell density (= total # of templates / total # of input cells), overlap & similarity	The clonal structure of T cells in blood and normal breast is more similar than between blood and tumor,. "Public" sequences (shared across patients) were relatively rare and likely to be immune background rather than tumor-specific.	Observed higher clonality (dominant clones) and increased T cell density in tumors vs. normal tissue that could be used as diagnostic biomarker.	2017	PNAS	Beausang *et al.* [33]
Cervix	CC / 25 + CIN / 30 + 20 HDs	Peripheral blood (from CC, CIN and HDs) and paired tumor and sentinel LNs tissue (from 16 CC)	5' RACE (SMARTer kit)	Richness, diversity (Shannon's entropy), repertoire overlaps, clonotype motif similarity	Repertoires in CC and CIN were more similar than either group to HDs. Diversity in repertoire from sentinel LNs from CC patients is higher than in tumor.	Circulating TCR diversity decreased gradually from HDs → CIN → CC, and was lowest in advanced CC. A lower richness in the TCR repertoire of sentinel lymph nodes correlated with poor prognosis in CC patients.	2018	Frontiers in Immunology	Cui *et al.* [51]
Nasopharynx	NPC / 39 & 15 + other nasopharyngeal diseases / 39 + 33 HDs	Peripheral blood from 39 NPC patients + paired tumor and adjacent normal tissue from 15 additional patients	5' RACE (SMARTer kit)	U/T index (= # unique sequences/ total # of sequences), diversity (Shannon's entropy), overlap & similarity (Morisita-Horn index), Vβ gene usage	Identified shared, "public" NPC-associated clonotypes found in blood or tumor tissue across multiple patients.	A more diverse TCR repertoire and specific V gene patterns were identified in the peripheral blood of NPC patients relative to the controls. Lower diversity of the tumor repertoire and lower similarity with paired normal tissue were both significantly associated with worse OS. In blood, higher TCR diversity was associated with worse prognosis.	2018	Cancer Immunology, Immunotherapy	Jin *et al.* [56]
	NPC / 720 (discovery cohort) + 90+287=377 (validation cohorts): including NPC patients, EBV-positive/negative donors (at high/low risk for NPC)	Peripheral blood (+ tumor tissue for a subset of cancer patients)	Multiplex PCR (+scTCR analysis for a subset of patients and TCR reconstruction from mRNA for tumor samples)	Incidence of public TCRs, T-score classifier (=total count of how many of the 208 NPC-enriched CDR3β sequences are present in a specific TCR repertoire), antigen specificity analysis (using GLIPH2 and GIANA)	The NPC-enriched TCRs were found to react against both EBV and non-EBV tumor antigens.	The T-score, based on the 208-TCR signature, accurately distinguished NPC patients from both healthy and at-risk controls. Critically, it successfully identified individuals with early-stage NPC in a prospective cohort up to a year before clinical diagnosis, showing superior performance compared to the standard EBV VCA-IgA serology test. Moreover, a higher T-score in pre-diagnostic individuals was significantly correlated with a shorter time to clinical diagnosis.<br>The presence and abundance of T cells with the NPC-enriched TCR signature within the tumor microenvironment were strongly associated with prolonged patient survival.	2025	Cancer Cell	Zhang *et al.* [57]
Head & neck	HNSCC / 162	Baseline tumor tissue	ImmunoSEQ	Richness, diversity (Shannon entropy),	Identification of five categories in the tumor microenvironment based on immune/stromal composition:(i) cytotoxic,(ii) plasma cell-rich,(iii) dendritic cell-rich,(iv) macrophage-rich,and (v) immune-excluded. Exhaustion markers and PD-L1 are higher in cytotoxic group and correlated with increased TCR richness/clonality. Impact of smoking history; never smokers have greater richness and improved survival.	High TCR richness and TLS signature in cytotoxic/plasme cell-rich groups predict better survival and suggest higher likelihood of long-term immunotherapeutic benefit. Macrophage-rich and immune-excluded subtypes, low TCR richness and immune suppression are associated with worst overall survival.	2023	Cancer Research Communications	Secrier *et al.* [28]
Brain	Pediatric brain tumors (15 major histological types) / 996	Baseline tumor tissue	TCR data inferred from RNASeq	Richness, clonality (Clonal Expansion-Activation Index, CEI), V/J gene usage, TCR similarity clustering	Repertoire clonality was not associated with TMB. TCR similarity clustering and *in silico/in vitro* analysis identified putative tumor antigens (e.g., PRAME, ZNF560, SMC1B) recognized by expanded T cell populations.	Distinct TCR repertoires correspond to brain tumor entities/subtypes and HLA backgrounds, providing an "immunogenomic fingerprint" that may aid in diagnosis and risk stratification. TCR clonality (higher CEI = more clonal, less diverse response) is positively correlated with improved patient outcomes across and within tumor types.	2025	Science Translational Medicine	Raphael *et al.* [46]
Penis	PSCC / 22	Paired tumor and adjacent normal tissue	Multiplex PCR (from RNA, with specific primers)	Diversity (inverse Simpson index), clonality (1 - normalized Shannon entropy), similarity (Morisita–Horn index), clonal overlap (Jaccard index), specificity groups (large-scale motif clustering)	Low TCR repertoire overlap between tumor and normal tissue.	Lower TCR repertoire diversity and higher clonality were observed in PSCC tissues, as compared with normal tissues. TCR repertoire in PSCC correlated with clinicopathologic features; specifically, increased clonality and reduced diversity were observed in advanced tumors (tumors with poor differentiation, high stages, and larger sizes). Tumors with high TCR clonality (oligoclonal expansion, especially of exhausted CD8+ T cells expressing TIM-3 and LAG-3) may be associated with functional immune suppression and poor response to immunotherapy.	2024	Cancer Immunology, Immunotherapy	Zhang *et al.* [35]
Gastric	GC / 19 (with different disease stages)	Paired tumor and adjacent normal tissue	Multiplex PCR (in-house primers panel)	Richness, diversity (Shannon's entropy), variation index (see formula in the methods), integrative analysis of microarray data and TCR repertoire variation index using the network-based Clique Percolation Method	Diversity and frequency distribution of T-cell clones did not change during tumourigenesis.	The overlap of T-cell clones between lesions and adjacent tissues decreased during malignant progression --> Increased heterogeneity (degree of TCR repertoire variation) can be used as diagnosis of disease progression. Tissue-infiltrating TCR repertoire variation in combination with transcriptional modules can predict OS.	2017	Scientific Reports	Kuang *et al.* [66]

TCR, T-cell receptor; PCR, polymerase chain reaction; NSCLC, non-small cell lung cancer; SCLC, small cell lung cancer; HD, healty donor; COPD, chronic obstructive pulmonary disease; ADC, adenocarcinoma; TMB, tumor mutational burden; ML, machine learning; MSS-CRC, microsatellite stable colorectal cancer; DFS, disease-free survival; LN, lymph node; TNM, tumor, node, metastasis; GC, gastric cancer; HBV, hepatitis B virus; HCC, hepatocellular carcinoma; PBMC, peripheral blood mononuclear cell; TIL, tumor-infiltrating lymphocytes; HG(S)OC, high-grade (serous) ovarian cancer; PFS, progression-free survival; WES, whole exome sequencing ; OS, overall survival; HRD, homologous recombinant deficiency; CNV, copy number variation; MIBC, muscle-invasive bladder cancer; RFS, relapse-free survival; PDAC, pancreatic ductal adenocarcinoma; TCGA, the cancer genome atlas; CC, cervical cancer; CIN, cervical intraepithelial neoplasia; NPC, nasopharyngeal cancer; HNSCC, head and neck squamous cell carcinoma; TLS, tertiary lymphoid structure; PSCC, penile squamous cell carcinoma
