# Supplementary material for: Harnessing TCR repertoires: predictive insights and therapeutic monitoring in cancer immunotherapy
Source: Immunooncol Technol. 2025 Oct 1;28:101076. doi: 10.1016/j.iotech.2025.101076 (PMC12615767; doi:10.1016/j.iotech.2025.101076)
Supplement: Supplementary Table S2 [file mmc2.pdf]

Table S2 - Overview of studies linking TCR repertoire profiling to patient outcome following conventional cancer treatments

Cancer	Cancer type / patient	Treatment	Sample	TCR-Seq method	TCR repertoire characteristics	TCR repertoire analysis	Association with patient response to treatment	Publication year	Journal	Reference
Breast	Breast cancer / 19	NACT prior to surgery	Tumor samples before and after NACT (at surgery)	5' RACE (SMARTer kit)	Diversity (Simpson index), clonal composition, V/J gene usage	-	Patients achieving complete response and partial response showed greater clonal expansion of TILs compared to those with stable disease, or progressive disease. Noteworthy, the diversity of TCR was further reduced in the post-NACT tumors of CR patients. Associations were drawn between gene expression (e.g., PD-1, PD-L1, FoxP3) and clinical response.	2016	International Journal of Oncology	Park *et al.* [76]
	Breast / 26	Standard first-line chemotherapy	Pre- (26) and post-treatment (7/26) peripheral blood samples	5' RACE (SMARTer kit)	Pairwise distance metric (Morisita-Horn index), HEC (highly expanded clone) ratios, Vβ gene usage patterns, and CDR3 motifs	HEC ratios of CD8+ T-cell were higher for HER2+ patients than those of HER2- patients, and several Vβ and CDR3 motifs were preferentially used in HER2+ patients.	Pre-chemotherapy HEC ratio did not significantly correlate with clinical response, but in HER2+ subgroup only, there was a trend (not significant) toward higher HEC ratios in responders. Circulating CD8+ T-cell repertoires evolved during chemotherapy, and increased CD8+ T-cell repertoire heterogeneity (compared to baseline) was associated with better clinical response.	2018	Cancer Immunology, Immunotherapy	Lin *et al.* [74]
	Metastatic breast cancer / 20	Systemic therapy (chemotherapy and possibly targeted therapies)	Baseline peripheral blood samples + 1 month after treatment	ImmunoSEQ	Richness, clonality (Simpson index), repertoire overlap (Morisita's index) + analysis of CTCs	Patients with low CTC counts at baseline had higher rates of functional anti-TAA T-cell responses.	Higher Simpson clonality index at baseline indicates more dominant T-cell populations and correlated with favorable response when combined with low CTC numbers --> combining CTC counts and T-cell (TCR) immunity in the blood provides a composite biomarker with the potential to improve management of metastatic breast cancer patients. Highest TCR clonality ("clonal expansion") at follow-up was seen in patients with both low CTCs and good clinical response.	2022	Frontiers in Oncology	Muraro *et al.* [77]
	Breast cancer / 94	NACT (8x), prior to surgery (+ targeted therapy for some HER2+ patients)	Peripheral blood samples before each treatment cycles and prior surgery	5' RACE (SMARTer)	Diversity (Simpson and Shannon's entropy indexes), repertoire overlap (Morisita-Horn index), V/J gene usage, motif analysis	TCR repertoire diversity was associated with patient age and clinical stage: younger and earlier stage patients had higher baseline TCR diversity. The TCR repertoire also correlated with tumor HER2 status, demonstrating immune differences dependent on the tumor subtype.	Specific Vβ gene usages before therapy correlated with better response to NACT and were independent prognostic factors. Loss of TCR diversity after NACT cycles was associated with better clinical response and predicted pathological complete remission. CDR3 motif clones significantly more frequent and greater change in TCR repertoire were associated with patient response.	2022	JCO Precision Oncology	Cai *et al.* [75]
Colorectal	Metastatic CRC (mCRC) / 16	Conventional chemotherapy	Baseline peripheral blood samples + longitudinal samples post-chemotherapy	Multiplex PCR (iRepertoire)	Richness, diversity (Chao1 and Shannon's entropy) and specific clonotypes distribution	Longitudinal TCR repertoire profiling reveals immune repertoire stability and individual-specific characteristics. Most mCRC patients experienced a decrease in TCR repertoire diversity after chemotherapy.	Patients with a favorable response had a more important decrease in peripheral TCR diversity, which was associated with greater reductions in tumor markers and tumor size, while higher TCR diversity before therapy correlated significantly with improved progression-free survival, highlighting its potential as a prognostic biomarker.	2021	Frontiers in Immunology	Chen *et al.* [49]
	CRC / 29	Neoadjuvant chemotherapy	Peripheral blood samples pre-treatment	Multiplex PCR	Diversity (D50 value, Shannon entropy and Simpson index)	-	Higher pre-treatment TCR repertoire diversity in peripheral blood was significantly associated with a favorable response to therapy. The D50 value is the best choice among the three indicators for evaluating the prognosis of neoadjuvant therapy (compared to Shannon and Simpson index).	2025	Cancer Medicine	Ma *et al.* [47]
Ovarian	HGSOC / 30	10 underwent surgery (R0 = no residual disease) and 20 received neoadjuvant chemotherapy (10 NACT-ER = excellent response, 10 NACT-PR = poor response)	Primary and metastatic tumor obtained prior definitive surgery or chemo	ImmunoSEQ	Richness, clonality (derived from Shannon entropy), evenness (Gini coefficient), clonal relatedness (nucleotide similarity, fraction of unique nucleotide sequences related to the most frequent sequence by an edit sequence threshold), repertoire similarity (Bhattacharyya coefficient), TCR convergence (aggregate frequency of clones sharing a variable gene and CDR3 amino acid sequence)	Enrichment of specific TCR and patterns of mutual exclusivity/co-occurrence by clinical group. Higher TCR diversity (more unique productive sequences and less clonal relatedness and higher TCR convergence) was observed in the R0 group compared to the NACT groups. Positive correlations were found between clonal relatedness and neoantigens, copy number variations, and mutation load.	Greater TCR diversity and less clonal relatedness in both primary and especially metastatic sites were associated with optimal resectability (and thus, a more favorable prognosis).	2021	iScience	Lee *et al.* [73]
	HGSOC / 27	Chemotherapy followed by PARPi (22/27) or not (5/27, PARPi-untreated controls)	Baseline peripheral blood samples (pre-PARPi) + 1 and 3 months after PARPi initiation	Multiplex PCR (from gDNA, with specific primers)	Diversity (inverse Simpson's index), clonal abundance changes (high-frequency vs. low-frequency clones), repertoire overlap (Morisita-Horn index)	No association was detected between baseline TCR diversity and age, BRCA1/2 mutation status, line of PARPi, or remission status before PARPi. The stability of the TCR repertoire was higher in PARPi-treated than untreated patient.	An increase in TCR diversity after 3 months of PARPi was strongly associated with a longer PFS and with a better response rate. Responders showed a marked reduction in high-frequency TCR clones.	2023	Gynecologic Oncology	Shu *et al.* [72]
Lung	Advanced lung cancer / 72 (39 LUAD, 10 LUSC, 23 SCLC)	Chemotherapy or targeted therapy for some patients	Baseline peripheral blood samples + longitudinal samples post-treatment	5' RACE (SMARTer kit)	Richness, diversity (log10-transformed Shannon-Weiner index(ShannonDI)), clonal expansion	Chemotherapy did not significantly impact global TCR diversity in early treatment cycles in advanced lung cancer patients.	A higher circulating TCR diversity (pre-/post-treatment) was found to be associated with improved therapeutic effects in the LUAD targeted therapy group. Higher baseline circulating TCR diversity, as an independent prognostic marker, correlated with better prognosis (longer PFS).	2021	Oncoimmunology	Wang *et al.* [79]
Head & neck, anal, cervix	HPV16-driven malignancies: HNSCC / 6, ASCC / 6, and CC / 7	Standard-of-care CRT	Tumor and peripheral blood samples pre-treatment as well as blood samples post-treatment (6 & 12 weeks)	ImmunoSEQ	Richness, clonality (1 - Normalized Shannon index), clonal relatedness, sequence similarity (clustering using GLIPH2), repertoires overlap (Morisita index)	-	Higher pre-treatment intra-tumoral TCR diversity and more clustered TCR structures were observed in responders. At baseline, responders showed greater overlap between TIL and PBMC repertoires, and higher baseline abundance of HPV16-specific TILs. In responders, CRT triggered an increase peripheral TCR clonality and clonal relatedness (broader longitudinal sharing). Responders showed more baseline clustered structures of TCRs maintained post-treatment and displayed significantly more maintained clustered structures.	2023	Frontiers in Oncology	Nenclares *et al.* [69]
Nasopharynx	NPC / 40	All RT + some received chemotherapy + some targeted therapy	Paired peripheral blood samples pre- and post-treatment	5' RACE (SMARTer)	Richness, diversity (inverse Simpson index, Pielou's evenness), clonal overlap index, V/J gene usage	-	Pre-treatment, the repertoire was highly skewed, dominated by a few expanded clones and the overlap index (repertoire similarity among patients) was higher in non-metastatic than metastatic groups. Significant post-treatment decrease in TCR diversity only in patients who developed distant metastases --> greater reduction predicted poor distant metastasis-free survival. Treatment expanded some TCR clones in both groups but increased proportion of large clones (with maintained diversity) after treatment was higher for responding patients. Lower similarity between pre- and post-treatment repertoires in metastatic patients correlated with a worse prognosis.	2021	Cancer Immunology, Immunotherapy	Zhang *et al.* [70]
Rectal	Advanced rectal cancer / 67	Neoadjuvant CRT	Tumor samples pre- (67) and post-treatment (23/67 patients)	Multiplex PCR (iRepertoire)	Diversity, clonality, repertoire overlap (Morisita index)	No direct association of TCR diversity with mutational or neoantigen load except in hypermutated tumors.	Higher intratumoral TCR diversity before CRT predicted a better pathological response. Also, high pre-treatment CD8+ T-cell infiltration was independently associated with good response. Larger changes (lower overlap) in the TCR repertoire before and after CRT were correlated with improved recurrence-free survival.	2021	Cancer Immunology, Immunotherapy	Akiyoshi *et al.* [71]
Lymphoma	DLBCL / 92	Conventional chemotherapy (R-CHOP)	Tumor biopsies (LN) at diagnosis + 12 non-diseased LNs	ImmunoSEQ	Richness, diversity (Shannon's entropy), clonality (1 - Pielou's evenness), % of top 10 and top 100 most frequent clones	DLBCL tumors had a significantly narrower and more clonal TCR repertoire than non-diseased nodes. Higher overall TCR diversity (entropy) in the tumor correlated with elevated immune checkpoint gene expression.	Highly dominant (narrow/clonal) TCR repertoires in the tumor were correlated with inferior OS and PFS --> more diverse repertoire is associated with better prognosis. The poorest outcomes (and most pronounced clonal expansions) were observed in the EBV+ DLBCL subtype, which is associated with known immunogenic antigens and poor prognosis.	2017	Clinical Cancer Research	Keane *et al.* [78]

TCR, T-cell receptor; NACT, neoadjuvant chemotherapy; TIL, tumor-infiltrating lymphocyte; CR, complete response; CTC, circulating tumor cell; TAA, tumor-associated antigen; CRC, colorectal cancer; PCR, polymerase chain reaction; HGSOC, high-grade serous ovarian cancer; PFS, progression free survival; LUAD, lung adenocarcinoma; LUSC, lung squamous cell carcinoma; SCLC, small cell lung cancer; HPV, human papillomavirus; HNSCC, head and neck squamous cell carcinoma; ASCC, anal squamous cell carcinoma; CC, cervical carcinoma; CRT, chemoradiotherapy; PBMC, peripheral blood mononuclear cell; NPC, nasopharyngeal cancer; RT, radiotherapy; DLBCL, diffuse large B-cell lymphoma; R-CHOP, rituximab, cyclophosphamide, doxorubicin hydrochloride (hydroxydaunorubicin), vincristine sulfate (Oncovin), and prednisone; LN, lymph node; OS, overall survival; EBV, Epstein-Barr virus
