# Supplementary material for: Harnessing TCR repertoires: predictive insights and therapeutic monitoring in cancer immunotherapy
Source: Immunooncol Technol. 2025 Oct 1;28:101076. doi: 10.1016/j.iotech.2025.101076 (PMC12615767; doi:10.1016/j.iotech.2025.101076)
Supplement: Supplementary Table S3 [file mmc3.pdf]

Table S3 - Overview of studies evaluating TCR repertoire profiling as a predictor of response to immune checkpoint inhibitors

Cancer	Cancer type / patient	Treatment type	Sample	TCR-Seq method	TCR repertoire characteristics	Association with response	Publication Year	Journal	Reference
Melanoma	Melanoma / 21	aCTLA-4	Peripheral blood	Multiplex PCR	Diversity (number of clonotype in the top 25th percentile)	aCTLA-4 induces increase of the diversity (= reflected in the richness) in the blood. Improve overall survival correlates with high-frequency TCRs present at baseline (clonal repertoire) that persist over treatment.	2014	Science Translational Medicine	Cha *et al.* [93]
	Melanoma / 46	aPD-1	TIL	ImmunoSEQ	Diversity (Shannon entropy), clonality (1-normalized Shannon entropy)	Pre-treatment samples obtained from responding patients showed higher numbers of CD8-, PD-1- and PD-L1-expressing cells at the invasive tumour margin and inside tumours, with close proximity between PD-1 and PD-L1, and a more clonal TCR repertoire.	2014	Nature	Tumeh *et al.* [82]
	Melanoma / 12	aCTLA-4	Peripheral blood	Multiplex PCR (ImmunTraCkeR test)	Richness and evenness (=# of top rearrangements needed to reach 50% of total frequency / total # of rearrangements)	Patients who experienced clinical benefit from aCTLA-4 (ipi) had a higher degree of evenness (less clonal) and higher richness as a continuous variable compared to patients who did not achieve clinical benefit.	2015	Journal for ImmunoTherapy of Cancer	Postow *et al.* [92]
	Melanoma / 56	αCTLA-4 (Ipi) followed by αPD-1 for progressing patients	Tumor (FFPE) (pre-treatment, on-treatment and post-progression)	ImmunoSEQ	Diversity (Shannon entropy) and clonality, immune score (expression profiling for immune-related genes, correlated with TCR clonality)	No difference in TMB in pre-treatment tumors between Rs or NRs. Higher tumor TCR clonality before PD-1 therapy (prior and after CTLA-4 blockade) was significantly associated with response to PD-1 blockade (but not to CTLA-4 blockade). The combination of high TCR clonality, high mutational load, and low burden of copy number loss best stratified patients with clinical benefit from immunotherapy.	2017	Science Translational Medicine	Roh *et al.* [85]
	Melanoma / 20	aPD-1 (Nivo) + aCTLA4 (Ipi) as neoadjuvant or adjuvant	Baseline tumor tissue + peripheral blood at baseline and on-treatment	ImmunoSEQ	Clonality (degree of dominance), expansion pattern	Baseline tumors from patients who relapsed tended to have lower T cell infiltration, lower clonality, and lower MHC & PD-L1 expression, along with low IFN-γ gene signatures.	2018	Nature Medicine	Blank *et al.* [88]
	Melanoma / 20	aCTLA-4 (17/20) / (aPD-1 (3/20) --> # too low to present the results)	Peripheral blood pre-/post-therapy	Spectratryping	Clonality index (degree of repertoire restriction)	Both CD4+ and CD8+ blood T cells in melanoma patients showed significantly greater clonality compared to HDs, with restriction more pronounced in CD8+ cells. The repertoires in CD4+ and CD8+ blood T cells before CTLA4 blockade were significantly more restricted (more clonal) in patient with better objective responses at 24 weeks and survived longer.	2019	Frontiers Immunology	Arakawa *et al.* [98]
	Melanoma / 80	aPD-1, aCTLA-4	Peripheral blood	Muliplex PCR (TraCkeR)	DE50	In samples obtained prior to treatment initiation, low DE50 values (higher clonality) were predictive of a longer progression-free survival and good responses to PD-1 blockade, but, on the other hand, predicted a poor response to CTLA4 inhibition.	2019	Cancer Immunology Research	Hogan *et al.* [91]
	Melanoma / 82	aPD-1, aCTLA-4	Tumor tissue	ImmunoSEQ	Clonality (1-evenness)	Pretreatment tumor TCR clonality and neoantigen load were marginally associated with best response with nivo/ipi, but not with ipi/nivo.	2019	Cancer Immunology Research	Yusko *et al.* [83]
	Melanoma / 37 + interferon	aCTLA-4	Peripheral blood and TIL	ImmunoSEQ	Clonality (1-evenness), Morisita index	In the pretreatment tumor microenvironment, T-cell clonality was significantly different and greater in patients who achieved disease control versus those with non-disease control as best response to treatment. In examining T-cell clonality in the circulation PBMC, no significant associations were found in the pretreatment samples.	2019	Oncoimmunology	Khunger *et al.* [84]
	Melanoma / 122 TCGA cohort: 412 melanoma + others spanning >29 tumor types	aPD-1	Primarily pre-treatment tumor biopsies	RNA-seq data with computational TCR reconstruction (for pan-cancer public data)	Richness, diversity (Renyi index), clonality (1–normalized Shannon entropy), repertoire similarity (Morisita–Horn and Simpson) and CDR3 motif analysis	The diversity of the TCR of tumor infiltrating T-cell at baseline is prognostic in various cancers, whereas the TCR clonality of T cell infiltrating metastatic melanoma pre-treatment is predictive for activity and efficacy of PD1 blockade immunotherapy.	2021	Nature Communications	Valpione *et al.* [63]
Lung	NSCLC / 40	aPD-1	Peripheral blood	Multiplex PCR	Diversity	Higher diversity of PD1+ CD8 cells correlates with better response to aPD-1.	2020	Cancer Immunology Research	Han *et al.* [95]
	NSCLC / 40	aPD-1 + platinum-based chemotherapy	Peripheral blood and tumor tissue at baseline and post-treatment	Oncomine (Ion-based)	Richness, diversity (Shannon entropy), eveness (Pielou's index), clonal space of top 1% clones,	Lower evenness (higher clonality) in pretreatment tissue was significantly associated with complete pathologic response. Higher top 1% clonal space in baseline tumor tissue was strongly associated with CPR, with high predictive accuracy (AUC ROC = 0.967, outperforming PD-L1 and TMB).	2021	Clinical Cancer Research	Casarrubios *et al.* [86]
	NSCLC /101	aPD-1 +/- chemotherapy	Peripheral blood	Multiplex PCR (Oncomine)	Diversity, Shannon entropy, evenness, convergence	For patients treated with aPD-1 (pembro) + chemotherapy, increased number of unique clones, increased Shannon entropy, and reduced evenness and convergence were associated with improved PFS, while only an increased number of unique clones were associated with improved OS. In contrast, in patients receiving aPD-1 as monotherapy, a reduced richness was associated with clinical benefit.	2023	ESMO Open	Abed *et al.* [94]
	Metastatic NSCLC / 119	aPD-1, aCTLA-4	Peripheral blood	ImmunoSEQ	Chao1, D50, inverse Simpson	Higher baseline T-cell diversity correlates with better therapeutic responses. Lower T-cell richness at the time of immune-related adverse events (irAEs) is observed in affected patients.	2024	Journal for ImmunoTherapy of Cancer	Altan *et al.* [97]
	NSCLC / 40	Chemoradiotherapy + aPD-L1	Peripheral blood	5'adapter ligation	Shannon-Weaver, inverse Simpson and Jaccard (clonal overlap) indices	Patients with a high diversity of CD8+PD-1+TCR repertoires, reflecting the host immune response, had a better response to CRT followed by aPD-1 (durvalumab) therapy.	2025	NPJ precision Oncology	Shirasawa *et al.* [96]

	NSCLC / 182	aPD-L1 / 9	Tumor tissue	RNAseq	Gini index	In a checkpoint inhibitor-treated NSCLC patient cohort, high TCR clonality was associated with therapy response and prolonged survival.	2025	BioRxiv	Yu *et al.* [32]
Gastrointestinal	GI / 143	aPD-1 +/- VEGFR-2 inhibitor	Peripheral blood	Multiplex PCR	Shannon entropy, repertoire similarity (Morisita index)	In the anti-PD-1 monotherapy cohort, patients with higher baseline TCR diversity exhibited a significantly higher disease control rate and a longer progression-free survival and overall survival than those with lower diversity.	2021	Clinical and Translational Oncology	Ji *et al.* [103]
	GI / 31	aPD-1	Peripheral blood	Multiplex PCR (Oncomine)	Shannon entropy, evenness	Compared to non-responders, the DE50 scores were significantly higher (= higher diversity) in responders. Patients with a high DE50 score showed better progression-free survival than those with a low DE50 score.	2024	Tranlational Gastroenterology and Hepatology	Wu *et al.* [102]
Head and neck	HNSCC / 41	aEGFR, aPD-1	Peripheral blood	ImmunoSEQ	Clonality (Simpson index)	Patients who achieved CR and PR had an increased TCR sequence diversity in their baseline samples, this tendency being more pronounced in HPV-negative patients or those with a smoking history.	2022	Journal for ImmunoTherapy of Cancer	Wang *et al.* [100]
Pancreas	Pancreatic cancer / 57	aCTLA-4 + GVAX / GVAX + listera monocytogene + aPD-1	Peripheral blood	ImmunoSEQ	Clonality and Morisita index	Long term survivors had significantly lower baseline clonality than short term survivors in the anti–CTLA-4 study. Long term survivors in the anti–PD-1 study had similar baseline clonality.	2018	JCI insight	Hopkins *et al.* [99]
Esophagus	ESCC / 19	Radiotherapy and aPD-1	Peripheral blood and tumor tissue	Multiplex PCR	Shannon entropy, repertoire similarity (Morisita index), clonality	Objective responsive patients had lower baseline intratumoral clonality compared with non-responsive patients. Furthermore, a landmark analysis revealed that patients with high baseline TCR diversity in peripheral CD8+ T cells had better OS and PFS.	2022	Oncoimmunology	Yan *et al.* [90]
Kidney	Metastatic clear cell renal cell carcinoma / 15	aPD-1 (Nivolumab)	Multipleregion tumor biospies (pre- and post-treatment) and matching peripheral blood		Clonality, repertoire similarity, clonotype clustering	Significantly higher number of expanded TCR clones (higher clonality) pre-treatment in responders' tumors suggesting pre-existing immunity.	2021	Cancer Cell	Au *et al.* [87]
Mesothelium	Mesothelioma / 23	aPD-L1	Tumor tissue	Multiplex PCR	Richness, Shannon entropy, evenness, clonality, d50 index	Patients with tumor TCRs evenness of <0.030 had a notably shorter PFS compared to those with evenness of ≥0.030. The clonality of patients in the partial response subgroup was significantly lower than that of patients with progressive disease.	2024	Thoracic Cancer	Nie *et al.* [89]
Urothelial	Urothelial cancer / 29	aPD-L1	Peripheral blood	ImmunoSEQ	Shannon entropy, clonality	Patients with durable clinical benefit displayed a higher proportion of TIL. Pretreatment peripheral blood TCR clonality below the median (thus higher diversity) was associated with improved PFS and OS.	2017	Plos Medicine	Snyder *et al.* [101]

TCR, T-cell receptor; PCR, polymerase chain reaction; CTLA-4, cytotoxic T-lymphocyte antigen 4; PD-1, programmed cell death protein 1; TIL, tumor-infiltrating lymphocyte; Ipi, ipilimumab (aCTLA-4); Nivo, nivolumab (aPD-1); TCGA, the cancer genome atlas; NSCLC, non-small cell lung cancer; CPR, complete pathologic response; Pembro, pembrolizumab (aPD-1); PFS, progression-free survival; OS, overall survival; PD-L1, programmed death-ligand 1; CRT, chemoradiotherapy; FFPE, formalin-fixed paraffin-embedded; TMB, tumor mutational burden; ICI, immune checkpoint inhibitor; HNSCC, head and neck squamous cell carcinoma; EGFR, epidermal growth factor receptor; PDAC, pancreatic ductal adenocarcinoma; GI, gastrointestinal; VEGFR-2, vascular endothelial growth factor receptor 2; ESCC, esophageal squamous cell carcinoma
