# Supplementary material for: Harnessing TCR repertoires: predictive insights and therapeutic monitoring in cancer immunotherapy
Source: Immunooncol Technol. 2025 Oct 1;28:101076. doi: 10.1016/j.iotech.2025.101076 (PMC12615767; doi:10.1016/j.iotech.2025.101076)
Supplement: Supplementary Table S4 [file mmc4.pdf]

Table S4 - Overview of studies assessing TCR repertoire profiling for early detection of response after immune

Cancer	Cancer type / patient	Treatment type	Sample	TCR-Seq method	TCR repertoire characteristics	Effect of therapy	Publication Year	Journal	Reference
Skin	Melanoma / 46	aPD-1	Tumor tissue	ImmunoSEQ	Diversity (Shannon entropy), clonality (1-normalized Shannon entropy)	Comparison of the TCR clonality at baseline and post-dosing biopsies showed that in samples from the response group, more than ten times as many clones expanded after anti-PD-1 therapy than in the progression group.	2014	Nature	Tumeh *et al.* [82]
	Melanoma / 21	aCTLA-4	Peripheral blood	Multiplex PCR	Diversity (number of clonotype in the top 25th percentile)	aCTLA-4 induces increase of the diversity (= reflected in the richness) in the blood (in general). Improve overall survival correlates with high-frequency TCR present at baseline that persist over treatment (maintenance of high clonality).	2014	Science Translational Medicine	Cha *et al.* [93]
	Melanoma / 13	aPD-1	Tumor tissue	5'RACE	Simpson index	The proportion of TCR-β clonotypes with frequency of >0.5% tend to be increased in the post-treatment tumor tissues of responders, compared with those of non-responders --> repertoire analysis revealed oligoclonal expansion of TILs in the tumor tissues of the responders.	2016	Oncoimmunology	Inoue *et al.* [105]
	Melanoma / 68	aPD-1	TIL	ImmunoSEQ	Richness and evenness	Temporal changes in intratumoral TCR repertoire upon treatment revealed expansion of T cell clones in the setting of neoantigen loss in patients with response.	2017	Cell	Riaz *et al.* [104]
	Melanoma / 23	aPD-1 (Nivo) monotherapy vs aPD-1 + aCTLA4 (Ipi) combination	Peripheral blood and tumor tissue pre- and post-treatment	ImmunoSEQ	TIL density, richness, clonality (1 - normalized Shannon entropy)	Rs to the monotherapy (aPD1) displayed an increase of clonal TILs and richness of the infiltrate. Combination therapy induced an increase in tumor-resident T cell clones in the peripheral blood (suggesting new recruitment or expansion from periphery).	2018	Nature Medicine	Amaria *et al.* [108]
	Melanoma / 82	aPD-1, aCTLA-4	Tumor tissue	ImmunoSEQ	Clonality	At week 13 post-treatment, individuals in either arm (ipi/nivo or nivo/ipi) that showed increased T-cell fraction and TIL clonality were 30 times more likely to achieve a best clinical response at week 33 than those without evidence of change.	2019	Cancer Immunology Research	Yusko *et al.* [83]
	Melanoma / 37	aCTLA-4	Peripheral blood and TIL	ImmunoSEQ	Clonality (1-evenness), Morisita index	Early on-treatment (4 weeks), there was a significant decrease in T cell clonality that was associated with improved overall survival and progression-free survival. In addition, analysis of temporal changes in tumor-infiltrating lymphocytes (TIL) and peripheral TCR repertoire revealed that responders had significantly higher clonal expansion of TIL in the circulation at 4 weeks than non-responders.	2019	Oncoimmunology	Khunger *et al.* [84]
	Melanoma / 20	aCTLA-4 (17/20) / (aPD-1 (3/20) --> # too low to present the results)	Peripheral blood pre-/post-therapy	Spectratryping	Clonality index (degree of repertoire restriction)	CTLA-4 blockade tended to increase repertoire restriction in CD4+ T cells post-treatment (increase clonality), consistent with pharmacologic expansion of diverse CD4+ clones. Increasing numbers of restricted TCR Vb-gene families and distinct peaks of CD8+ blood T cells from pre- to post-immunotherapy were correlated with significantly decreased survivals (increased clonality associated with poorer survival).	2019	Frontiers Immunology	Arakawa *et al.* [98]
	Melanoma / 55	aPD-1, aPD-1 + aCTLA-4	Peripheral blood	RNAseq	Number of expanded clonotype (>0.5%)	Responding patients have larger clones (those occupying >0.5% of repertoire, increased clonality) for both aPD-1 and aPD-1 + aCTLA-4 post-treatment than non-responding patients or controls, and this correlates with effector memory T cell percentage.	2020	Nature Medicine	Fairfax *et al.* [106]
Lung	NSCLC / 21	aPD-1	Peripheral blood and tumor tissue (pre- and post-treatment)	ImmunoSEQ (on 9/21 patients)	Shared clones between blood and tumor, clonality	After PD-1 blockade, there was systemic expansion of mutation-associated, neoantigen-specific T-cell clones in the peripheral blood, especially in responders; some of these were undetectable pre-treatment, indicating therapy-induced priming/expansion. The frequency of T-cell clones shared between tumor and peripheral blood increased after treatment (i.e., infiltration into, or release from, the tumor), especially in major responders. Higher TIL clonality (post aPD-1) and greater shared clones between tumor and periphery were found in tumors with major pathological response.	2018	The New England Journal of Medicine	Forde *et al.* [109]
	NSCLC / 40	aPD-1	Peripheral blood	Multiplex PCR	Diversity and clonality	Increased clonality in peripheral blood during treatment in aPD-1 responders.	2020	Cancer Immunology Research	Han *et al.* [96]
	NSCLC / 21	aPD-1	Serial peripheral blood (pre-treatment, 2, 4 weeks and at surgery post-treatment) + tumor tissue at surgery (pre- and post-ICI)	ImmunoSEQ	Richness, clonality, similarity (Jaccard index), clonal expansion	Post-treatment tumor, but not normal lung tissue, from patients with major pathologic response (MPR) had a significantly higher T-cell clonality relative to patients with non-MPR.	2020	Clinical Cancer Research	Zhang *et al.* [110]
	NSCLC / 19	aPD-1 (Pembro) + chemo (only for 6/33 patients)	Peripheral blood at baseline and after treatment	Multiplex PCR (Oncomine)	Richness, evenness (similarity of clone size, Pielou's index), convergence (% of clones identical in aas but different in nts), Jaccard similarity index, V/J gene usage	Increased peripheral richness during treatment was associated to greater clinical benefit. Patients with greater Jaccard similarity index values between pretreatment and various treatment timepoints showed improved PFS. High baseline usage of TRBV20-1 was predictive of durable clinical benefit and improved survival.	2021	Cancers	Dong *et al.* [113]
	NSCLC / 71	aPD-L1	Peripheral blood	Multiplex PCR	Clonality, TCR convergence	Though patients' TCR repertoire diversity showed mixed responses to the treatment, patients exhibiting increased diversity on day 15 attained significantly longer overall survival.	2021	Cancer Immunology, Immunotherapy	Naidus *et al.* [112]
Pancreas	Pancreatic cancer / 57	aCTLA-4 + GVAX GVAX + listera monocytogene + aPD-1	Peripheral blood	ImmunoSEQ	Clonality and repertoire similarity (Morisita index)	The majority of patients receiving these treatments experience a net diversification of their peripheral TCR repertoires. Second, patients receiving aCTLA-4 experienced larger changes (increase in diversity) in their repertoires, especially in combination with GVAX. Long term survivors in the anti–PD-1 study had similar baseline clonality; however, significantly higher clonality was observed after 3 cycles of treatment.	2018	JCI Insight	Hopkins *et al.* [99]
Kidney	Kidney cancer / 25	aPD-1	Peripheral blood and tumor tissue	Multiplex PCR	Diversity and clonality	Diversity index for PBMC TCRA and TCRB were significantly decreased 1 month after treatment in responders versus non-responders. Similarly, patients with increased TCR clonality indicated a superior OS and PFS compared with those with decreased TCR clonality. Responders exhibited a significantly higher number of expanded clones than those of non-responders, 1 month after treatment. Importantly, these expanded clones were significantly sustained, 3 and 6 months after treatment initiation in responders.	2021	Oncoimmunology	Kato *et al.* [107]
Glioblastoma	Glioblastoma / 30	aPD-1	TIL	Multiplex PCR	Richness, Shannon entropy	Increased clonal T cell diversity (both richness and Shannon entropy) following treatment with neoadjuvant nivolumab compared to control group (treated with standard-of-care therapy). Significant association between the overall number of TCR clonotypes post-treatment and survival --> higher richness is associated with longer PFS.	2019	Nature Medicine	Schalper *et al.* [111]
Head and neck	Head and Neck / 38	aPD-1 / aPD-1+aCTLA4 / aPD-1 + aLAG-3	Tumor tissue	Single cell (10x Genomics)	Inverse Simpson index	aPD-1+aLAG-3, but not aPD-1+aCTLA-4, induces widespread TCR sharing among the different transcriptional states, as well as increased TCR diversity in responding patients.	2025	Cancer Cell	Li *et al.* [115]
Urothelial	Urothelial cancer / 29	aPD-L1	Peripheral blood	ImmunoSEQ	Shannon entropy, clonality	Patients with durable clinical benefit demonstrated more substantial expansion of tumor-associated TCR clones (increased clonality) in the peripheral blood 3 weeks after starting treatment.	2017	Plos Medicine	Snyder *et al.* [101]
Esophagus	ESCC / 19	Radiotherapy and aPD-1	Peripheral blood and tumor tissue	Multiplex PCR	Shannon entropy, repertoire similarity (Morisita index), clonality	While analyzing on-treatment TCR diversity, responsive patients had significantly higher TCR diversity in the peripheral CD8+ T cells compared with non-responsive patients.	2022	Oncoimmunology	Yan *et al.* [90]

Lymphoma	Classical Hodgkin lymphoma (cHL) / 56 + 11 untreated cHL patients + 17 HDs	aPD-1 (nivolumab)	Peripheral blood at baseline and during treatment	ImmunoSEQ (only matched samples on 20/56 patients)	Diversity (Shannon entropy), clonal expansion (fold change post/pre), singleton vs non-singleton clones (expansion of new vs pre-existing clones)	Significant expansion of singleton clones (clones not detected or detected at minimal levels at baseline) was observed during nivolumab therapy, especially in patients achieving a complete response. CD4+ T cell repertoire diversity significantly increased on treatment, most notably among complete responders; CD8+ T cell diversity did not change significantly.	2020	Nature Medicine	Cader *et al.* [114]

TCR, T-cell receptor; CTLA-4, cytotoxic T-lymphocyte antigen 4, PD-1, programmed cell death protein 1; PCR, polymerase chain reaction; TIL, tumor-infiltrating lymphocytes; Nivo, nivolumab (aPD-1); Ipi, ipilimumab (aCTLA-4); NSCLC, non-small cell lung cancer; PFS, progression-free survival; ESCC, esophageal squamous cell carcinoma
