# Supplementary material for: Harnessing TCR repertoires: predictive insights and therapeutic monitoring in cancer immunotherapy
Source: Immunooncol Technol. 2025 Oct 1;28:101076. doi: 10.1016/j.iotech.2025.101076 (PMC12615767; doi:10.1016/j.iotech.2025.101076)
Supplement: Supplementary Table S5 [file mmc5.pdf]

Table S5 - Overview of studies assessing TCR repertoire profiling for predictive or early detection of response to cellular therapies, cancer vaccines, or ither non-ICI treatments

	Cancer type / patient	Treatment type	Sample	TCR-Seq method	TCR repertoire characteristics	Effect of therapy	Publication Year	Journal	Reference
Cellular therapies	PDA / 31 + metastatic melanoma / 32	Autologous tumor-infiltrating lymphocytes **preclinical research, assessing the in vitro expansion mechanisms*	Baseline peripheral blood and tumor tissue + the infusion product	5' RACE	Diversity (Shannon entropy) and clonality (inverse of normalized entropy), top25 TCR tracking, repertoire overlap	*Ex vivo* expansion drastically alters the TIL TCR repertoire - most clones are lost and rare clones in the tumor overgrow in the final produt. The degree of dominant clone maintenance varies by patient, tumor, and cancer type. Expansion capacity is mainly determined by intrinsic properties of T-cell clones (exhaustion for ex.). Different tumor fragments from the same patient may differ in their TCR repertoires, and replicated expansions from spatially distinct fragments can result in very different TIL compositions --> the loss of tumor-dominant (and potentially tumor-reactive) TCRs during expansion may reduce therapeutic efficacy, as overgrown *in vitro*–fit clones may lack tumor reactivity.	2020	Clinical Cancer Research	Poschke *et al.* [124]
Cellular therapies	GBM / 9	Expansion of GILs **preclinical research, assessing the feasibility of using GILs as cellular therapy*	Baseline tumor tissue and expanded GILs	ImmunoSEQ	Diversity (Simpson index), clonality, repertoire overlap and clone tracking	*In vitro* expansion induces a dramatic increase in repertoire clonality, with the low-frequency or marginal clones *in situ* becoming predominant after culture --> expansion bias is determined by pre-existing transcriptional state: higher cytotoxicity/activiation and proliferation, while selective expansion is mostly antigen-independent in GBM. CD4+ T cells preferentially outgrow CD8+ T cells in standard expansion protocols.	2022	Neuro-Oncology Advances	Lu *et al.* [125]
Cellular therapies	Ovarian cancer / 3	Expansion of TILs **preclinical research (2 different protocols compared)*	Baseline tumor tissue and expanded TILs (with high dose IL-2 or low dose IL-2 +aCD3/CD28)	Multiplex PCR	TRBV gene usage profiling, clonality (poly or clonal dominance)	Expansion method decisively shapes TIL TCR repertoire: mitogenic stimulation (low-dose IL-2 + aCD3/CD28) yields a more even, polyclonal distribution of TRBV usage among both CD4+ and CD8+ populations. This protocol preferentially “rescues” or expands T cells that do not proliferate well under standard high-dose IL-2 only conditions. Standard high-dose IL-2 leads to outgrowth of a restricted set of dominant clones (bias toward specific TRBV families), indicating clonal selection and possible loss of overall TCR diversity. Both CD4+ and CD8+ T cells can be robustly expanded by either method.	2022	Journal of Immunological Methods	Kongkaew *et al.* [123]
Cellular therapies	Advanced gastric cancer / 19	Expanded PBMCs (following DC-CIK protocol)	*Ex vivo* product (PBMCs) at day 0 (pre-) and day 15 post-expansion	ImmunoSEQ	Diversity (Shannon index), clonality, evenness, unique vs shared clonotypes	Patients with a larger decrease in TCR diversity (post/pre) and greater increase in clonality had better clinical outcomes. A high reduction in unique clonotypes after expansion correlated with improved survival. Patients whose *ex vivo* expansion led to a higher proportion of CD8+PD-1+ T cells experienced significantly longer OS and PFS than those below this threshold.	2022	American Journal of Cancer Research	Wang *et al.* [119]
Cellular therapies	Metastatic melanoma / 13	Autologous tumor-infiltrating lymphocytes	Peripheral blood and tumor tissue pre- and post-treatment + the infusion product	Multiplex PCR (SEQTR method) for bulk TCRSeq and 10x Genomics for scTCR-Seq	Richness, diversity (Shannon entropy), clonality (1 - Pielou's index), repertoire overlap, clone tracking	Baseline tumors from responders are enriched for tumor-reactive clonotypes, which preferentially expand and dominate ACT products in these patients. Non-responders’ ACT products, by contrast, were more likely to be composed of blood-borne (not tumor-resident) T cells. A much higher absolute number and greater diversity of tumor-reactive CD8+ TCRs is transferred and engrafted in responders than non-responders. Reprogramming of TILs in the infusion product, which lose their exhaustion state and acquire a more effector/memory phenotype.	2024	Immunity	Chiffelle *et al.* [116]
Cellular therapies	NSCLC / 14	Autologous tumor-infiltrating lymphocytes	Peripheral blood and tumor tissue pre- and post-treatment + the infusion product	ImmunoSEQ (+10x Genomics for scTCR-Seq of the infused product and baseline tumor)	Clone tracking, repertoire overlap	Non-response to TIL therapy was associated with a rapid loss of tumor-reactive and neoantigen-specific T-cell clonotypes in both peripheral blood and tumor tissue after TIL administration. In addition, acquired immune escape (such as loss of target neoantigen or HLA allele in tumor cells) mirrored the loss of matching T-cell clonotypes, linking repertoire decay to immunoediting and tumor evolution.	2025	Nature Cancer	Wang *et al.* [122]
Cellular therapies	mCRC / 1	Enriched TILs targeting a KRAS mutation (G12D)	Peripheral blood and tumor tissue pre- and post-treatment (a progressing/responding lesion post-treatment) + the infusion product	ImmunoSEQ	Clonotype identification and frequency tracking in different samples	The *in vivo* persistence of the 3 clonotypes found in the product was different. The robust initial response (regression of all metastatic lesions) was associated with high-frequency, polyvalent T-cell engraftment. Nonetheless, a single lesion later progressed due to immune evasion via loss of the target HLA allele, not due to loss of the KRAS mutation itself.	2016	The New England Journal of Medicine	Tran *et al.* [117]
Cellular therapies	B-cell malignancies (ALL, NHL, CLL) / 10	CD19 CAR T cells	The infusion product + post-ACT sorted CAR T cells at multiple timepoints	ImmunoSEQ for bulk TCR-Seq and 10x Genomics for scTCR-Seq	Diversity (Shannon entropy), repertoire overlap (Morisita index), clone tracking, clone kinetic patterns	CAR-T infusion products were initially highly polyclonal, but clonal diversity declined post-infusion, with a few clones expanding markedly and others contracting or disappearing (infused clones showed variable abilities). Some of the expanding clones present early after infusion remained highly represented even at "very late" timepoints, while others did not persist. Expanding/persistent clones mostly originated from clusters in the infusion product with higher expression of genes related to cytotoxicity and proliferation.	2020	Nature Communications	Sheih *et al.* [118]
Cellular therapies	Refractory multiple myeloma / 11	BCMA-specific CAR T cells	Bone marrow samples at 3 timepoints: pre-treatment, D28 and 3M post-infusion	ScTCR-Seq with 10x Genomics	Clone size (hyperexpanded, large, medium, small, single), diversity (Shannon index), clone tracking	Higher TCR diversity at baseline predicts longer response. Pretherapy T-cell exhaustion and clonal hyperexpansion equals poor outcome. After CAR T infusion, some clones expanded, some contracted, and new clones emerged. Residual or relapsing tumor at 3 months often showed new dominant TCRs, reflecting ongoing immune dynamics and/or tumor evolution. Preferential expansion of TCF1+ CD27+ (stem-like) T cells post-therapy in all patients, but persisting abundance is associated with longer responses.	2022	Blood Cancer Discovery	Dhodakpar *et al.* [121]
Cellular therapies	Metastatic synovial sarcoma / 12	Engineered CD4+/CD8+ T cells with affinity-enhanced NY-ESO1/LAGE-1a-specific TCR	The infusion product + peripheral blood and tumor tissue (for some patients) post-treatment	ImmunoSEQ	Diversity (Shannon entropy), clonality (inversed normalized entropy), clone tracking	Responders had higher expansion and longer persistence of NY-ESO1-specific TCRs than non-responders, which retained functional cytotoxicity. Long-lived clonotypes showed maintenance of clonal diversity without contraction, and persistent clones were never found only in one T-cell subset. The infused product was dominated by effector memory cells; however, the *in vivo* persisting pool shifted toward progenitor-like central memory and stem-cell memory subsets within a month and was stably maintained in this form long-term.	2018	Cancer Discovery	D'Angelo *et al.* [120]

	AML / 6 + 3 HDs	HSCT	Bone marrow samples	5' RACE	TCR specificity prediction (ML models were constructed to recognize WT1-specific TCRs), public and private "cluster" TCR analysis, frequency and diversity of WT1-specific TCRs	AML patients in complete remission after HSCT have a more diverse, polyclonal, and widely distributed repertoire of WT1-specific TCRs than patients with relapse or healthy controls. The majority of identified WT1-specific TCR clusters were found only in AML patients and not in HDs, supporting the use of such cluster signatures as highly relevant for leukemic immune monitoring. The presence and diversity of WT1-specific TCRs after HSCT may reflect the strength of the graft-versus-leukemia (GVL) immune effect and potentially identify patients at lower risk for relapse, or those who might benefit from further immunotherapeutic intervention.	2025	Annals of Hematology	Gielis *et al.* [126]
**Beyond ICIs and cellular therapies**	Prostate cancer / 5	Sipuleucel-T	Peripheral blood pre- and post-treatment and tumor tissue post-treatment	ImmunoSEQ	Diversity (Shannon Entropy), TCR commonality (Baroni-Urbani and Buser index)	Cancer patient have a greater diversity of their repertoire at baseline compared to healthy donors. Sipuleucel-T reduced blood TCR diversity but increased diversity in resected prostate tissue. Furthermore, Sipuleucel-T increased TCR sequence commonality between blood and cancer tissue in treated versus untreated patients.	2016	Cancer Research	Sheikh *et al.* [131]
	Glioblastoma / 15	Lysate-pulse DC vaccine	Peripheral blood and tumor tissue	ImmunoSEQ	Richness, overlapp frequency	Higher degrees of overlap between TIL and blood TCRs or the development of an increased overlap following immunotherapy, was correlated with improved clinical outcome.	2017	Cancer Immunological Research	Hsu *et al.* [135]
	Melanoma / 3	Neo-antigen DC vaccine	Peripheral blood	ImmunoSEQ	Clonotype frequency	Neo-antigen DC vaccine increase frequency of most existing prevacine TCRb clonotype and revealed previously undetected clonotypes for all six neoantigen.	2015	Science	Carreno *et al.* [130]
	Melanoma / case report	CSF-470 vaccination	Peripheral blood	ImmunoSEQ	D25, D50	Increase in oligoclonality was observed in the peripheral T-cells immune repertoire throughout immunaization with CSF-470. Tumor specific clonotypes were still detectable in blood 2 years after immunization.	2018	Frontiers in Immunology	Aris *et al.* [132]
	Melanoma / 21	ICIs + NEO-PV-1	Peripheral blood	Multiplex PCR	Diversity, clonality	Tumor antigen specific TCR were expanded following vaccination.	2020	Cell Reports Medicine	Poran *et al.* [133]
	Malignant pleural mesothelioma / 9	DC-based vaccine	Peripheral blood	Multiplex PCR	Chao index	DC vaccine induces expansion of PD1+CD8+ population	2020	Journal of Immunotherapy of Cancer	Vroman *et al.* [134]

ICI, immune checkpoint inhibitor; TCR, T-cell receptor; PDA, pancreatic ductal adenocarcinoma; TIL, tumor-infiltrating lymphocyte; GBM, glioblastoma; GIL, glioma-infiltrating lymphocyte; IL-2, interleukin-2; PCR, polymerase chain reaction; PBMCs, peripheral blood mononuclear cells; DC-CIK, dendritic cell-cytokine induced killer cell; OS, overall survival; PFS, progression free survival; ACT, adoptive cell transfer; NSCLC, non-small cell lung cancer; HLA, human leukocyte antigen; mCRC, metastatic colorectal cancer; ALL, acute lymphoblastic leukemia; NHL, Non-Hodgkin lymphoma; CLL, chronic lymphocytic leukemia; CAR, chimeric antigen receptor; AML, acute myeloid leukemia; HD, healthy donor; HSCT, hematopoietic stem cell transplantation; ML, machine learning; DC, dendritic cell
